# An Overview of NO Signaling Pathways in Aging

**DOI:** 10.3390/molecules26154533

**Published:** 2021-07-27

**Authors:** Ali Mohammad Pourbagher-Shahri, Tahereh Farkhondeh, Marjan Talebi, Dalia M. Kopustinskiene, Saeed Samarghandian, Jurga Bernatoniene

**Affiliations:** 1 Medical Toxicology and Drug Abuse Research Center (MTDRC), Birjand University of Medical Sciences, Birjand 9717853577, Iran; ali.pourbagher.shahri@gmail.com; 2 Cardiovascular Diseases Research Center, Birjand University of Medical Sciences, Birjand 9717853577, Iran; farkhondeh2324@gmail.com; 3 Faculty of Pharmacy, Birjand University of Medical Sciences, Birjand 9717853577, Iran; 4 Department of Pharmacognosy and Pharmaceutical Biotechnology, School of Pharmacy, Shahid Beheshti University of Medical Sciences, Tehran 1991953381, Iran; talebi.m@sbmu.ac.ir; 5 Institute of Pharmaceutical Technologies, Faculty of Pharmacy, Medical Academy, Lithuanian University of Health Sciences, Sukileliu Pr. 13, LT-50161 Kaunas, Lithuania; DaliaMarija.Kopustinskiene@lsmuni.lt; 6 Noncommunicable Diseases Research Center, Neyshabur University of Medical Sciences, Neyshabur 9318614139, Iran; 7 Department of Drug Technology and Social Pharmacy, Faculty of Pharmacy, Medical Academy, Lithuanian University of Health Sciences, Sukileliu Pr. 13, LT-50161 Kaunas, Lithuania

**Keywords:** nitric oxide, nitric oxide synthase, aging, senescence, NO signaling pathways, therapeutic agents

## Abstract

Nitric Oxide (NO) is a potent signaling molecule involved in the regulation of various cellular mechanisms and pathways under normal and pathological conditions. NO production, its effects, and its efficacy, are extremely sensitive to aging-related changes in the cells. Herein, we review the mechanisms of NO signaling in the cardiovascular system, central nervous system (CNS), reproduction system, as well as its effects on skin, kidneys, thyroid, muscles, and on the immune system during aging. The aging-related decline in NO levels and bioavailability is also discussed in this review. The decreased NO production by endothelial nitric oxide synthase (eNOS) was revealed in the aged cardiovascular system. In the CNS, the decline of the neuronal (n)NOS production of NO was related to the impairment of memory, sleep, and cognition. NO played an important role in the aging of oocytes and aged-induced erectile dysfunction. Aging downregulated NO signaling pathways in endothelial cells resulting in skin, kidney, thyroid, and muscle disorders. Putative therapeutic agents (natural/synthetic) affecting NO signaling mechanisms in the aging process are discussed in the present study. In summary, all of the studies reviewed demonstrate that NO plays a crucial role in the cellular aging processes.

## 1. Introduction

Nitric oxide (NO) is one of the main signaling molecules in the body that shows its principal performance in an unconventional manner. NO exerts its effects on several molecular targets and can regulate various functions such as neurotransmission, vascular tone, transcription of genes, translation of mRNA, and protein post-translational modifications [1]. NO can react with superoxide anion (O_2_^−^), resulting in the formation of potent oxidant peroxynitrite (ONOO^−^), and subsequently interacting with biomolecules such as proteins, lipids, and DNA via direct oxidation reactions or indirect radical-mediated mechanisms [2,3,4,5,6]. ONOO^−^ is responsible for many pathological processes in mammalian organelles including cytotoxicity induction, oxidation, protein modifications, lipids peroxidation, DNA damage, cell death, mitochondrial disruption, dysregulation of signal transduction, and apoptosis [7]. Besides, various methods are available for the synthesis of ONOO^−^. ONOO^−^, as a highly reactive molecule, can result in the generation of oxidizing and nitrating species [6]. ONOO^−^ and its decomposition yields containing NO_2_, CO^−^, and OH can impair several reactions comprising the tyrosine nitration of proteins, the inactivation of superoxide dismutase (SOD), and tissue damage [8]. ONOO^−^ induced nitrosative stress has the capacity to induce the appearance of breaks in single-strand DNA, which subsequently activates the poly-ADP-ribose polymerase (PARP) [9]. NO is produced in mammals by three distinct forms of NO synthase (NOS), coded by three distinct genes: neuronal ‘n’NOS (or NOS-I), inducible ‘i’NOS (or NOS-II), and endothelial ‘e’NOS (or NOS-III). All NOS proteins are homodimers [10,11,12]. Furthermore, in mammals, NO can also be formed, resulting in NOS-independent pathways, explicitly by consecutive reduction of nitrate (NO_3_^−^) and nitrite (NO_2_^−^). NO_2_^−^ has the capability to be univalently reduced to NO through the transition of metal-comprising enzymes, e.g., deoxymyoglobin/deoxyhemoglobin (deoxyHb/deoxyMb), and xanthine oxidase (XO) at the time in which the partial pressure of oxygen (*p*O_2_) levels is low. The aforementioned NOS-independent gates for NO production represent the NO_3_^−^/NO_2_^−^/NO pathway or O_2_-independent formation of NO [13]. NO signaling, NO donors, and NOS inhibitors are very important in the pathophysiology of age-related diseases and their associated putative therapeutic approaches. In this study, we review the role of NO in the aging processes of cells.

## 2. Mechanisms of NOS

The common substrate for all NOS enzymes is l-arginine. NOS enzymes simultaneously bind multiple cofactors and prosthetic groups: heme, glutathione, molecular oxygen, reduced nicotinamide-adenine-dinucleotide phosphate (NADPH), flavin adenine dinucleotide (FAD), flavin mononucleotide (FMN), (6r-)-tetrahydro-l-biopterin (BH_4_), and Ca^2+^ calmodulin [1]. NOS enzyme uses the flavins FAD and FMN to transfer the electrons from the carboxy-terminal reductase domain of NADPH to the amino-terminal oxygenase domain of heme. The transferred electrons are used to reduce and activate O_2_ and oxidize l-arginine to l-citrulline and NO. NOS catalyzes two-step oxidation of l-arginine: firstly, l-arginine is hydroxylated to *N*-hydroxy-l-arginine, which subsequently is oxidized to l-citrulline and NO [14,15]. Binding sites of BH_4_ and l-arginine are located at the cysteine ligand of the heme and the oxygenase domain. The latter is the binding site for molecular oxygens as well [16,17].

**Figure 1:**
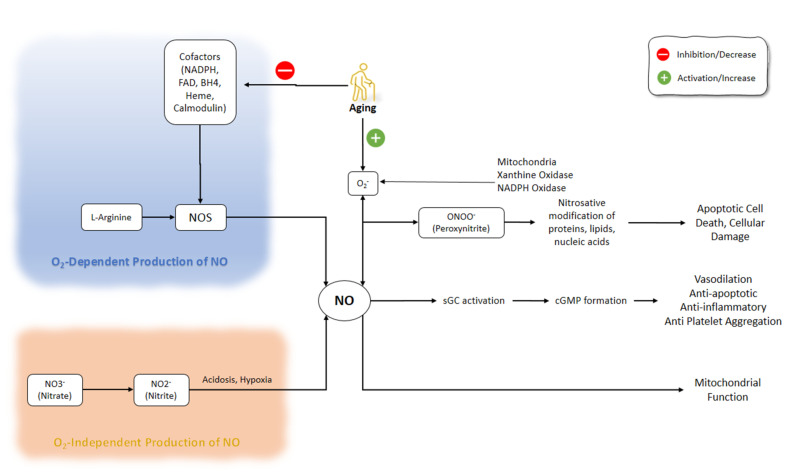
NO production pathways and NO functions. There are several critical steps in the production of NO that might be O_2_-dependent or O_2_-independent. Inhibition of NOS cofactors is important in the aging process. Besides, aging is accompanied by the activation of superoxide. NO: Nitric Oxide; NOS: Nitric Oxide Synthase; NADPH: nicotinamide-adenine-dinucleotide phosphate; FAD: flavin adenine dinucleotide; BH_4_: (6r-)-tetrahydro-l-biopterin.

The process of electron transfer from NADPH to the heme in nNOS and eNOS is facilitated by the binding of calmodulin. The elevation of intracellular Ca^2+^ activity is a key factor responsible for the increased affinity of calmodulin to NOS. However, even at low levels of intracellular Ca^2+^ concentrations, calmodulin-binding with iNOS does not change because of the distinct amino acid composition of the calmodulin-binding site [18,19,20]. NO affects several enzymes and proteins, including the activation of soluble guanylyl cyclase and the production of cyclic guanosine monophosphate (cGMP) [1]. Regulation of the function of smooth muscle by cGMP, which is associated with the mediation of phosphodiesterase (PDE) isozymes, plays a part in smooth muscle relaxation [21]. NO production pathways and NO functions are shown in (Figure 1).

### 2.1. nNOS

Ca^2+^ and calmodulin control neuronal NOS (nNOS) activity. The subcellular distribution and function of nNOS were determined by the direct association of the PDZ domain of nNOS with other proteins [22]. PDZ domain brings up to an area that entails 80–120 amino acid residues that perform as components taking part in protein-protein interactions [22,23]. The canonical PDZ domain of nNOS connects to the nNOS adaptor protein NOS1AP, also well known as carboxy-terminal PSD-95-Dlg-ZO1 [PDZ] ligand of nNOS (CAPON). The ternary complex made by PDZ domain interactions between *N*-methyl-d-aspartate (NMDA) receptor, PSD-95, and nNOS performs the action of scaffolding nNOS to the NMDA receptor, and NMDA-encouraged Ca^2+^ influx is consequently well coupled to the activation of nNOS and the subsequent formation of NO [24]. The nNOS has been found in various parts and tissues of the body, including the brain, spinal cord, sympathetic ganglia, adrenal glands, peripheral nitrergic nerves of the nervous system, as well as epithelial cells of different tissues such as kidney macula densa cells, pancreatic islet cells, and vascular smooth muscle cells. The most abundant presence of nNOS has been determined in the mammal skeletal muscle [25].

nNOS does not seem to be involved in the acute neurotransmission in the central nervous system (CNS); however, it can mediate the long-term regulation of synaptic transmission (long-term potentiation, long-term inhibition). Furthermore, nNOS is involved in the central regulation of blood pressure. This has been shown by the induction of systemic hypertension while blocking nNOS activity in the medulla and hypothalamus [1]. The effects of nNOS on the regulation of vascular tone are independent of its CNS effects. The production of NO by nerves that contain nNOS (nitrergic nerves) stimulates NO-sensitive guanylyl cyclase and decreases the tone of the innervated smooth muscles in the periphery including blood vessels [25,26]. Ca^2+^/calmodulin (CaM)-dependent protein kinases, cyclic AMP-dependent protein kinase (PKA), cGMP-dependent protein kinase, and protein kinase C (PKC) participate in the phosphorylation of nNOS [27].

The effects on the smooth muscles of the corpus cavernosum and the relaxation of nitrergic nerves affects penile erection [28,29]. The relaxation of these smooth muscle cells is mediated by cyclic GMP, which can be degraded by phosphodiesterases (predominantly isoform 5). Therefore, a residual nNOS activity is needed for the selective PDE5 inhibitors to be effective [30]. 

### 2.2. iNOS

iNOS enzymes are exclusively located in macrophages and their production is triggered when needed. The bacterial lipopolysaccharides and cytokines can influence the expression of iNOS [25,31]. When iNOS is expressed, it is activated continuously and is not affected by the intracellular Ca^2+^ concentrations. The iNOS in macrophages excretes large amounts of NO, which is important for them as a toxic defense molecule [32]. NO can disrupt the enzymes containing iron in their catalytic centers, such as iron-sulfur cluster-dependent enzymes (complexes I and II) entailed in mitochondrial electron transport, ribonucleotide reductase (the rate-limiting enzyme in DNA replication), and *cis*-aconitase (a key enzyme in the citric acid cycle) [32,33]. Recently, studies on ^13^C tracing and mitochondrial respiration revealed a new observation on the modulation in NO-mediated macrophage metabolic programming attributed to the regulation of the tricarboxylic acid (TCA) cycle and the accumulation of itaconate with a great fractional combination of glucose carbon at the overhead of glutamine carbon, which could link the suppression of pyruvate dehydrogenase (PDH) and aconitase to NO. NO has a functional impact on the accumulation of metabolite hallmarks of M1 polarization, including citrate, succinate, and itaconate. Indeed, during the stimulus, glucose uptake was elevated, immune-response gene-1 (Irg1) was upregulated, and citrate and itaconate were produced, incorporating glucose-derived carbon. [34,35,36]. At higher concentrations, NO can directly impede target cell DNA and affect strand breaks and fragmentation [37,38]. In addition to macrophages, other non-immune cells can produce NO by iNOS upon induction via cytokines and thus can affect other cells, i.e., endothelial cells exert NO-mediated lysing of tumor cells and cytokine-induced iNOS in hepatocytes synthesize NO to kill malaria sporozoites [39,40].

### 2.3. eNOS

eNOS is the leading source of NO in vascular endothelium. Its promotor expression exists in various types of cells such as cardiomyocytes, platelets, specific brain neurons, in syncytiotrophoblasts of the human placenta, and in LLC-PK_1_ kidney tubular epithelial cells [1,25]. NO production is regulated by Ca^2+^-activated calmodulin as the increase of intracellular Ca^2+^ induced binding of calmodulin subsequently increases the eNOS activity [19]. Other factors can also regulate the activity of eNOS, e.g., heat shock protein 90 (hsp90) acts as an allosteric modulator that promotes the eNOS’ (re)coupling and therefore its activity [41,42]. Another regulator of eNOS is caveolin-1 [43]. It is a tonic inhibitor interacting with the eNOS localized in caveolae [44]. The lack of caveolin-1 results in enhanced endothelium-dependent relaxation in mice [45]. The inhibitory effect of caveolin-1 can be blocked by the presence of calmodulin and hsp90 [46]. Fluid shear stress can activate eNOS independent of intracellular Ca^2+^ increase through phosphorylation of eNOS at several serine (Ser), threonine (Thr), and tyrosine (Tyr) residues [47,48]. During the regulation of eNOS activity by phosphorylation, the probable changes in phosphatase activity are able to have an impact on NO generation. Certainly, protein phosphatase 1 (PP1) and protein phosphatase 2A (PP2A) play diverse roles in the regulation of eNOS phosphorylation [48]. According to recent studies, aging is considered to be related to the greater protein acetylation levels and the defeat of sirtuin-1 deacetylation activity, which could subsequently cause the reduced eNOS activity [49,50]. Four lysine residues are found within the cAMP-response element-binding protein (CREB)-binding protein (CBP) of eNOS. The identification of two of these residues as a consequence of the acetylation suggests that SIRT1 may target more than a single residue within this domain, regulating enzymatic activity via the modulation of deacetylation of the targeted residues. Moreover, by knowing the recognized role of the phosphorylation in eNOS regulation, in a similar manner to other proteins, the acetylation and phosphorylation might both be responsible for the regulation of eNOS activity [51].

## 3. Aging and NO Signaling

Aging can negatively affect cellular functions even in the absence of obvious disease. However, the disorders emerge with aging in several systems and organs including the cardiovascular system, CNS, skin, kidneys, thyroid, male and female reproductive system, muscles, and immune system. The underlying mechanisms of these age-related disorders are complex and involve multiple pathways, factors, and cellular targets which are beyond the scope of this review. Herein, we made an in-depth focus on the involvement of NO signaling pathways in the complex scenario of aging-related disorders. The disturbed NO signaling contributes to a variety of aging pathologies. In a broad sample (763 participants, age range of 19–107 years), Montesanto et al. (2013) explored the genetic diversity in human NOS genes and found the association between decreased cognitive function and physical performance with nNOS rs1879417 and iNOS rs2297518 polymorphism, respectively [52].

### 3.1. Cardiovascular Aging and NO

The impairment of the vascular function due to endothelial dysfunction is one of the main heart and vasculature alterations during cardiovascular aging. The endothelial dysfunction is characterized by the impaired vasodilator reaction to the flow or the agonists and one of its fundamental causes is the decreased bioavailability of NO under pathological conditions such as atherosclerosis, hypertension, and hypercholesterolemia [53,54,55,56]. The reduced abundance or activity of eNOS, the increased levels of endogenous NOS inhibitors, and the reduced supply of l-arginine, or the increased NO degradation or scavenging, can result in decreased NO bioavailability.

As part of the urea cycle, hydrolyzing l-arginine to ornithine and urea by arginase can affect the bioavailability of l-arginine. NO performance increases through the iNOS in the macrophages following the inhibition of arginase [57]. Arginase can also modulate the vascular tone by regulating l-arginine supply for eNOS activity. Berkowitz et al. (2003) have shown that arginase activity and expression are upregulated in the aorta rings of old rats while the NOS activity and cGMP levels and, consequently, the endothelial activity, were reduced. Moreover, arginase inhibition increased vasodilation in the aortic rings of old rats compared with young adult rats. Furthermore, arginase inhibition restored NOS activity and cyclic GMP levels in the old rat vessels compared with those of young rats. Arginase competitively controlled the bioavailability of l-arginine for eNOS by limited NO production despite the increased expression of eNOS or adding exogenous l-arginine. The pretreatment with arginase inhibitors was sufficient to restore l-arginine responsiveness in the old rat vascular ring preparations. This result indicates that the enzyme kinetics strongly favors arginase. During arginase inhibition, l-arginine can restore cytosolic NOS responsiveness and thus restore vasorelaxation. Arginase was in contact with NOS, contributing to impaired relaxation of the endothelium. In brief, Berkowitz et al.’s (2003) research showed that arginase expression and activity increases in the vasculature with advancing age and leads to endothelial dysfunction [58].

Chou et al. (1998) found lower expression and activity of eNOS in spontaneously hypertensive rats (SHR) compared with non-SHR at both young and old age. The SHR developed the early onset of the decrease in eNOS expression and activity. However, the eNOS decline did not progress by aging in a way that eNOS activity was lower in the old non-SHR rats compared to the old SHR rats. The iNOS expression and activity showed inversed correlation to eNOS in both SHR and non-SHR. The iNOS expression was increased further by lipopolysaccharide (LPS) stimulation only in the 14–17- and 63-week groups of SHR. Hypertension control could regulate the abnormal iNOS expression and activity in SHR but, a “cause and effect” relationship was not established. Besides, the increase in levels of the tumor necrosis factor-alpha (TNF-α) and NO_2_^−^/NO_3_^−^ induced by LPS was much higher in SHR than in non-SHR at both groups of 14–17- and 63-week [59]. In the studies of Wu et al. (1999), the significant elevation was observed in basal plasma and aorta levels of NO_2_^−^ in SHR treated with LPS model. In vitro, the ACh-induced relaxation was decreased in the aortae isolated from SHR. However, this dissimilarity between the SHR and non-SHR was eliminated after the treatment of the rings with nitro-l-arginine methyl ester (l-NAME), and the damage of NO production was observed in the SHR. Expression of iNOS in both groups treated with LPS was augmented. These data supported the hypothesis that the raised plasma NO level in SHR might be the result of the NO release from protein-bound dinitrosyl nonheme iron complexes (DNIC) in the vascular bed, leading to the abolished hypertension [60]. Vaziri et al. (1998) concluded that the l-arginine/NO pathway was upregulated in young SHR before and after the onset of hypertension, which showed that NO formation was augmented in young SHR both before and after the commencement of the hypertension [61]. Further studies have demonstrated that the elevated production of NO in SHR, which was additionally improved by LPS treatment, is related to a primary expression of iNOS [62]. 

Cernadas et al. (1998) showed that aged rats had an abnormal hypotensive response to acetylcholine and bradykinin and a retained hypotensive response to nitroprusside sodium. Furthermore, the pressure effect of l-arginine antagonist (l-Nv-nitro-l-arginine) caused an increased sensitivity response and angiotensin II caused a decreased vasoconstrictor response in aged rats. The latter was improved after inhibition of NOS. Aged rats had higher plasma levels of nitrite and nitrate, higher cGMP content, and elevated eNOS and iNOS expression in the aortas. However, the activity of eNOS was significantly decreased in aged rats compared to the young ones, these results correlated with the iNOS activity. The locally released cytokines from endothelial injury sites in the vascular wall might have induced iNOS expression. The iNOS-related NO could decrease the action of the eNOS, thereby favoring impaired endothelium-dependent vasorelaxation. On the other hand, during aging, iNOS activity could play a significant function in retaining the vascular tone [63].

The reduction in endothelial vasoreactivity and excessive diastolic relaxation is linked with cardiovascular aging. Zeiman et al. (2001) showed that l-arginine increased ventricular relaxation in both young and aged rat hearts. The increased myocardial NOS-cGMP signaling was correlated with the elevated levels of eNOS protein in cardiac endothelial cells of aged rats. Furthermore, aged rats had elevated calcium-dependent NOS activity. However, in isolated myocytes, there was no difference between NOS activity and protein abundance. Administrating l-arginine decreased baseline isovolumic relaxation and left ventricular end-diastolic strain in both young and aged rat hearts. Sodium nitroprusside (a NO donor) promoted the NO/cGMP mediating pathway, which was attenuated by the soluble guanylyl cyclase inhibitor 1*H*-[1,2,4]oxadiazolo-[4,3-*a*]quinoxalin-1-one [64]. In the studies of the role of soluble guanylyl cyclase (sGC) in aging, it was found that sGC was a heterodimeric enzyme composed of one α1 and β1 subunit that shared a molecule of heme. It was observed that the existence of heme depended on the expression of enzymatic activity and rendered sGC sensitivity to NO. In numerous examples, the activation of sGC and production of cGMP mediated the impact of NO. Moreover, in the vasculature, NO-induced relaxation and repression of platelet aggregation happened through cGMP [65].

Regarding the preclinical findings, asymmetric dimethylarginine (ADMA) is an important endogenous NOS inhibitor in aging-associated cardiovascular risk. In aging-related biological conditions such as telomere shortening and cell senescence, ADMA is implicated [66]. Concentrations of vitamin D have reverse interactions with ADMA concentrations while h-sensitivity C-reactive protein (hs-CRP) has direct interactions with ADMA concentrations [67]. In a study by Scalera et al. (2006), it was observed that l-arginine could avert the commencement of endothelial aging in ADMA or the homocysteine-treated cells’ elevation of the production of NO and, subsequently, the induction of heme oxygenase (HO-1) enzyme activity and protein expression [68].

Vascular endothelial cells produce and release endothelin-1 (ET-1), a potent vasoconstrictor protein [69]. In stable, older, and sedentary adult humans, ET-1 signaling was enhanced, which can cause tonic vasoconstriction in peripheral arteries [70,71,72]. Furthermore, aortic endothelial cells of older individuals have shown a higher synthesis of ET-1 relative to young adult donors with distinct pathologies [73].

Donato et al. (2009) found an inverse relationship between decreased endothelium-dependent dilation and increased expression of the endothelial cell ET-1 protein. Moreover, the endothelium-dependent dilation was suppressed by the ET-1 signaling in old mice compared to the young. In endothelial cells collected from brachial arteries and peripheral veins of healthy young and old men, reduction in neither eNOS nor activated serine 1177 phosphorylated isoform (PeNOS) of eNOS was found. These data suggested the contribution of ET-1 expression and bioavailability, but not eNOS, to the vascular endothelial dysfunction in aging [74].

In vessels of aged animals, the decreased flow-induced dilation mainly depended on the compromised NO-mediated dilation [75,76]. Superoxide (O_2_^−^) increases within the vascular wall with aging, which decreases the bioavailability of NO, leading to endothelial dysfunction in aging [76,77,78]. Production of superoxide could increase the interruption of NO resulting in considerable vasomotor dysfunction, which was attributed to vascular aging [79,80]. Collectively, it was proposed that reaction of NO with O_2_^−^ led to accelerate the production of ONOO^−^, which attenuated the bioavailability of NO. Moreover, dysregulation of the eNOS caused impairment in the release of NO in the endothelium and increased superoxide formation [81]. This process of aging in the vascular wall can be worsened further as O_2_^−^ formation can also be improved by a decline in SOD function [82]. Sun et al. (2004) showed a decrease in the shear stress-induced dilation and NO release following aging in rat mesenteric arterioles and arteries. Moreover, the shear stress stimulation of NO synthesis was decreased in aged vessels, evidenced by the decrease in the absolute perfused nitrite (NO_2_^−^/NO_3_^−^) compared to the young vessels. Furthermore, the aged rats’ vasculature had lower SOD levels due to the decreased extracellular superoxide dismutase (ecSOD) expression, but not the Cu/Zn-SOD and Mn-SOD expression. Suppressed ECSOD expression further decreased the NO bioavailability following the response to the shear stress in the aged vessels. In summary, isolated mesenteric arteries have decreased NO-mediated vasodilation response to the shear stress due to the lower NO bioavailability following the increased O_2_^−^ production. The latter is caused by the suppressed ECSOD expression and consequently the lower SOD activities [83].

Caveolin, the intrinsic protein of caveolae, is quantitatively linked with the eNOS in ventricular myocytes and endothelial cells [84]. In the former, eNOS is linked with caveolin-3 and, to a lesser degree, caveolin-1, while, in endothelial cells, eNOS is associated only with caveolin-1 [85]. Arreche et al. (2012) found increased NOS activity, decreased eNOS, and caveolin-1 protein levels, as well as increased iNOS activity in the ventricle of the middle-aged rats [86]. The dissociation of caveolin-1 from eNOS correlated with aging. During the hemorrhage-induced hypovolemia, NOS activity and protein levels were increased in the myocardium while the caveolin-1 presence was decreased, which resulted in further dissociation of eNOS. The iNOS increased following blood loss in middle-aged rats. Thus, the modulatory roles of caveolin-1 on cardiac NOS have been demonstrated in the hypovolemic and aging states.

NO contributes to the regulation of water and electrolyte homeostasis in the cardiovascular system [87]. Arza et al. (2015) found that, following 1-month water restriction, both young and adult rats showed decreased NOS expression and activity related to the increased caveolin-1 levels. Interestingly, aging caused functional alterations in the cardiovascular system, evidenced by more remarkable ventricular NOS formation following water restriction and hypovolemia in adult rats. These findings demonstrate the roles of NO and its caveolin regulatory proteins in preserving cardiac physiological function during dehydration and aging [88]. 

Aging impairs the control of glucose metabolism, resulting in the decreased glucose tolerance and chronic hyperglycemia in the elderly. These changes can mimic accelerated aging and act as a risk factor for cardiovascular diseases [89,90,91]. Levels of NO and peroxynitrite might play a pivotal part in the progression of late impairment in the diabetic vasculature and kidney [92]. NO donor agents and the NO generated by iNOS persuaded insulin resistance via the S-nitrosation of proteins entailed in the primary stages of insulin action, for instance, insulin receptor β (IRβ), insulin receptor substrate 1 (IRS-1), and protein kinase B (Akt). Even though a number of studies have proposed that NO and iNOS might be important in numerous aging-associated complications, the contribution of NO pathways on aging-induced insulin resistance is indistinct [93]. Moreover, hyperglycemia along with aging correlated with endothelial dysfunction, vascular stiffening, and remodeling [94,95]. The basal activity of eNOS and endothelial dysfunction are evident at an advanced age and during hyperglycemia [96,97,98]. In addition, hyperglycemia has been shown to increase iNOS expression and activity, which correlates with excessive NO production and subsequent cellular damage [99].

Rogers et al. (2013) showed increased senescence markers following the glucose level fluctuations in human umbilical vein endothelial cells (HUVECs). The increase of aging markers was due to the elevated Akt’s transcription and expression and its downstream signaling targets. Moreover, eNOS production was decreased, which resulted in iNOS overexpression, decreased bioavailability of NO, and endothelial dysfunction [100].

Yu et al. (2011) reviewed the relationship between aging-induced heart failure and insulin resistance. The precise mechanism was not deceptive in this issue. The documented outcomes showed that insulin resistance might have adverse effects on bioenergetics and myocardial metabolism. Regarding the investigation of isolated perfused hearts obtained from aged rats and cardiomyocytes, the attenuation of contractile reaction and uptake of glucose to insulin, as well as the dampening of the Akt–eNOS–NO pathway due to the post-insulin receptor signaling, were perceived [101].

Classical pro-atherosclerotic stimuli such as oxidized low-density lipoprotein (oxLDL), proinflammatory cytokines, and reactive oxygen species (ROS) caused endothelial cell apoptosis and disrupted the endothelial monolayer integrity leading to vascular damage and atherosclerosis [102,103,104]. The endothelial NO production could inhibit apoptosis by suppressing caspases (a vital factor in apoptosis cascade) through the S-nitrosylation of its critical cysteine [105,106,107]. Hoffmann et al. (2001) reported a three-fold increase in levels of oxLDL, tumor necrosis factor-a-induced apoptosis, and caspase-3-like activity in aged HUVECs compared to the young cells. The substantially suppressed eNOS expression and decreased overall S-NO content implied that eNOS downregulation in aged HUVECs can lead to an age-dependent increase in vulnerability to apoptosis. This was confirmed as the knockdown of eNOS could elevate the apoptosis rate which was abolished by exogenous NO donors. Furthermore, the shear stress did not affect eNOS protein expression, S-NO content, and apoptosis rate in the aged HUVECs. Overexpression of wild-type eNOS restored the antiapoptotic effects of the shear stress but did not affect it when the shear stress was absent. Strikingly, apoptosis in aged HUVECs was further abrogated by the transfection of constitutively active phosphomimetic eNOS (S1177D) [108].

Oelze et al. (2104) showed that, in the absence of glutathione peroxidase-1 (GPx-1), the aging-associated production of oxidants, the activation of the redox system, and the presence of the dysfunctional and uncoupled eNOS are intensified. They found the increased phosphorylation of protein kinase C, protein tyrosine kinase, and eNOS S-glutathionylation associated with an uncoupling in aged peroxidase-1-deficient (GPx-1−/−) mice. The emerging role of GPx-1 ablation in aging animals has a considerable influence on the burden of oxidative damage, representing the experiential variation between vascular dysfunction in aged wild-type and GPx-1−/− mice [109]. GPx-1 is an intracellular antioxidant enzyme which has an important role in the reduction of H_2_O_2_ to H_2_O in order to delimitate its adverse impacts. GPx-1 is a selenocysteine-comprising enzyme that has been involved in the prevention and progression of numerous usual, chronic, and complicated diseases containing cancer, diabetes, and cardiovascular disease [110,111].

Sirtuins are a family of protein deacetylases that regulate aging processes. They depend on NAD^+^ and thus control cell metabolism along with other cellular functions. Sirtuin 3 (SIRT3) acts mainly by regulating mitochondrial bioenergetics, an essential prerequisite for healthy aging [112,113]. Lu et al. (2020) found that SIRT3 mRNA and protein are decreased in aged human and rat veins. Ad-SIRT3 gene transfer increased the expression and concentration of SIRT3, MnSOD, catalase (CAT), eNOS, and NO. Furthermore, it reduced the neointimal thickness and neointimal area/media area ratio [114]. Mattagajasingh et al. (2007) showed that SIRT1 contributed to the endothelium-dependent vasodilation by activating eNOS in the endothelium of rat aortic rings through deacetylation in a caloric restriction animal model. SIRT1 targeted lysines 496 and 506 in the calmodulin-binding domain of eNOS. Moreover, SIRT1 inhibition decreased NO bioavailability and thus endothelium-dependent vasodilation in arteries [51]. 

**Figure 2:**
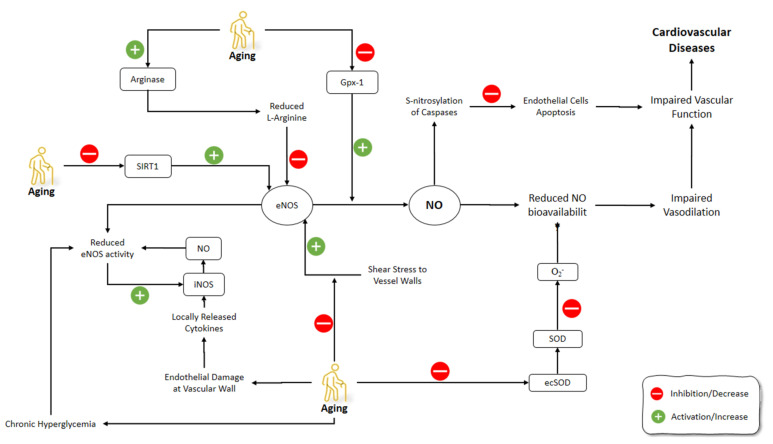
Effects of aging on NO pathways involved in the cardiovascular system. NO: Nitric Oxide; iNOS: Inducible Nitric Oxide Synthase; eNOS: Endothelial NOS; GPx-1: Glutathione peroxidase-1; ecSOD: Extracellular superoxide dismutase; SOD: superoxide dismutase.

Red blood cells (RBCs) affect NO generation and scavenging by hemoglobin-dependent nitrite. The changes in levels of NO_2_^−^ could regulate the NO hemostasis as it can be reduced to NO and also can be oxidated into NO_3_^−^ [115]. RBCs can affect vascular activity by modulating NO through suppressing NO_2_^−^ oxidation by their antioxidants [116,117]. It has been shown that aged RBCs have lower antioxidant levels [118]. In a study by Owusu et al. (2013), regulatory roles of RBC on vascular hemostasis of NO vasodilatory mechanisms were investigated. Older RBCs had higher NO_2_-oxidation kinetics and NO scavenging levels compared to young RBCs. The latter was reflected in the inhibited acetylcholine and NO-donor dependent vasodilation in isolated aortic rings. The similarity of NO_2_^−^ reduction levels between young and old RBCs was confirmed by the inhibition of nitrite-dependent vasodilatation under oxygenated and deoxygenated conditions in old RBCs [119] (Figure 2).

### 3.2. CNS Aging and NO

**Figure 3:**
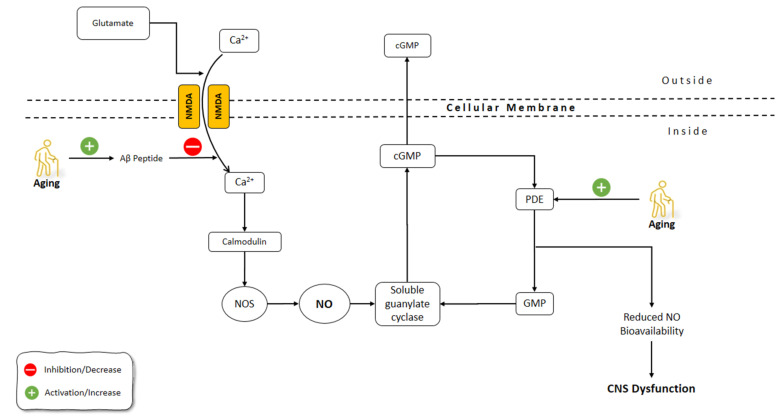
Effects of aging on NO pathways involved in CNS functions. NO: Nitric Oxide; NOS: Nitric Oxide Synthase; Aβ: Amyloid-beta; GMP: guanosine monophosphate; cGMP: Cyclic guanosine monophosphate; Pde: phosphodiesterase; CNS: Central Nervous System; NMDA: *N*-methyl-d-aspartate receptor.

The brain undergoes morphological and functional alterations during aging. The burden of oxidative stress on the CNS is more prominent due to the relative deficiency in the antioxidant defense mechanisms of CNS [120]. CNS aging is greatly affected by oxidative stress, which involves the production of free radicals including superoxide (O_2_^−^), hydrogen peroxide (H_2_O_2_), NO, and peroxynitrite (ONOO^−^) [121,122]. Reactive oxygen species (ROS) containing O_2_^−^, H_2_O_2_, and the OH^−^, and reactive nitrogen species (RNS) including ONOO^−^ and NO, are very important in the pathophysiology of neurodegenerative diseases. Due to the great reactive impact of ROS, they chemically interact with various biological molecules, causing significant alterations in cellular tasks, an increased probability of spreading to regions with high metal levels, and induced neuronal death [123,124,125]. Several functions of the CNS are related to NO, including neurotransmitter release, synaptic plasticity, and the regulation of neuronal electrical activity [126,127]. It has been shown that NO has a prominent role in aging processes and related disorders of CNS [128,129] (Figure 3).

Uttenthal et al. (1998) observed that in the cortex of the aged rats, nNOS-immunoreactive multipolar neurons were similar to those of the young rats in the terms of quantity, but had an abnormal morphology: varicose, vacuolated, and fragmented structure with an irregular outline and loss of spines. In addition, neurons that were weakly nNOS-positive with a ring of immunoreactive cytoplasm could be found only in the aged rat cortex. On the other hand, iNOS-immunopositive neurons were found only in the aged rat cortex. The old rat cortex had lower nitrotyrosine-positive cells (a biomarker of oxidative stress) compared to the total NOS-positive neurons, while the opposite was observed in the young rat cortex. The formation of nitrotyrosine-containing tissue proteins has the potential to modulate NO and O_2_^−^ concentrations related to the introduction of protein tyrosyl residues to reaction. Other significant elements that might contribute to the greater protein nitrotyrosine levels identified could be an augmented production of O_2_^−^ and an abridged rate of turnover of the related tissue proteins with age. According to the outcomes obtained in the study, the increase in nNOS and iNOS expression in the aged rat cortex is not essentially related to the proportional increase in NOS [130]. In a study by Hilbig et al. (2002), a decrease of nNOS expression was observed during the aging process in rats [131].

Brain aging has been linked with mitochondrial dysfunctions [132]. According to recent studies, NOS is not present in the mitochondrial matrix but rather makes part of the outer membrane. Lores-Arnaiz and Bustamante (2011) found a decrease in mitochondrial NO production in both synaptic and non-synaptic neurons of the aged rat cortex. Furthermore, the impairment of mitochondrial functions: ATP deficiency, H_2_O_2_ production at high levels, abnormal calcium homeostasis, and decreased levels of NO were observed in the aged rat compared to the young rat cortex neurons [133]. 

Synaptic mitochondria, which need to sustain the energy required for synaptic activity, showed present functional differences when compared to non-synaptic mitochondria [134]. However, although the aging-induced changes in the brain are thought to be mediated by the increased activity of NOS [135], the experimental data is rather controversial, as the demonstrated results range from an increase to a significant reduction in the nNOS activity in aged rat or mice models [136]. Mitochondrial investigations of NO effects support the hypothesis that NO levels could be increased during aging: endogenously-produced NO can inhibit mitochondrial respiration at cytochrome oxidase with the Ki ~50 nM (physiological range of NO concentrations is 0.1–100 nM) [137,138]. Moreover, NO can irreversibly suppress mitochondrial respiration, increase the production of ROS and RNS, and induce the mitochondrial permeability transition pore [137,138].

In the CNS, glutamate activates the *N*-methyl-d-aspartic acid (NMDA) receptor, which facilitates the influx of Ca^2+^ into the cell. The binding of Ca^2+^ to calmodulin activates the NOS production of NO which further activates the soluble guanylate cyclase and production of cGMP [139]. One of the main causes of dementia development is the aggregation of amyloid β (Aβ) peptides [140,141]. It has been shown that Aβ peptide interferes with the calmodulin-dependent NO synthesis. Chalimoniuk and Strosznajder (1998) showed that with advanced age, phosphodiesterase degradation of cGMP increased, which resulted in the decreased basal levels of cGMP. Moreover, the inhibition of cGMP-phosphodiesterase did not restore the NMDA-induced production of cGMP in the hippocampus and cerebellum of aged rats. However, the brain cortex in aged animals responded to the inhibition of cGMP-phosphodiesterase and showed a preserved production of cGMP. Interestingly, the NOS activity in the aged brain hippocampus and cerebellum was elevated by 175 and 160%, respectively. The treatment with Aβ fragment, peptide 25–35, reduced the Ca^2+^ transfer mediated by NMDA and thus calmodulin-dependent NO formation. The elevated NOS activity, despite disturbed cGMP-dependent signaling in the hippocampus and cerebellum of aged animals, might affect learning and memory function during aging, and the accumulation of Aβ during aging might be the cause of these disorders [142]. Activity-related Ca^2+^/calmodulin-dependent protein kinase II (CaMKII) autophosphorylation and AMPA receptor phosphorylation were reported as having crucial implications for hippocampal dentate long-term potentiation. Interruption in these mechanisms could directly associate with Aβ-induced impairments in memory and hippocampal synaptic plasticity [143,144].

Under chronic neuroinflammation conditions such as brain aging or neurodegenerative diseases, excessive levels of NO can be found at the choroid plexus (CP) via iNOS mediation, an epithelial layer that forms the blood-cerebrospinal fluid barrier (BCSFB). Glial cells are the source for these NO when they are chronically activated under the conditions such as aging or neurodegeneration [145,146,147]. Leukocytes reduce the neuroinflammation by entering CSF through the CP gateway; however, leukocytes do not always enter the CSF optimally during neuroinflammatory conditions. Thus, increased levels of leukocytes in the CSF can act as a potential therapeutic strategy [148,149,150,151,152,153,154]. It was suggested that NO alone in pathologic levels did not lead to neuronal death and gliosis. NO together with other cytotoxic and pro-inflammatory factors could induce neurodegeneration [147]. 

Baruch et al. (2015) showed that NO suppressed the leukocyte trafficking in choroid plexus (CP) by inhibiting epithelial nuclear factor-κB (NF-κB) p65 nuclear translocation in AD-Tg mice. Furthermore, the administration of the NO scavenger, rutin, enhanced the levels of leukocytes in the brain [155]. 

**Figure 4:**
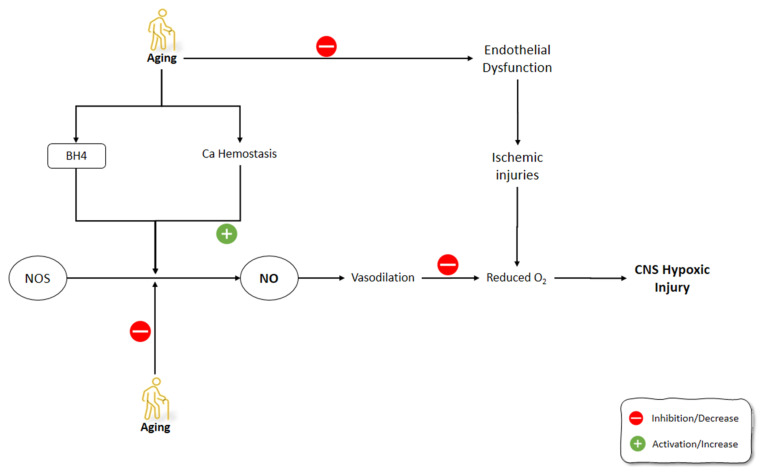
Effects of aging on NO production mechanisms and signaling pathways involved in the development of CNS ischemic injuries. NO: Nitric Oxide; NOS: Nitric Oxide Synthase; BH4: (6r-)-tetrahydro-l-biopterin.

The tolerance of the brain towards hypoxemic conditions reduces by aging [156]. One of the suggested mechanisms for this phenomenon was the increase of free radicals during hypoxia and even at the reoxygenation period [157]. Hypoxic/ischemic conditions, including stroke, aneurysm, trauma, and infection, are among the most common causes of CNS dysfunction [158]. The increased frequency of hypoxic conditions during aging, along with the dysfunctional activity of the aged brain during these situations, highlight the importance of understanding the underlying mechanism behind these malfunctions. It has been shown that, contrary to eNOS and nNOS, which are constitutively expressed, iNOS induction is enhanced under conditions such as hypoxia [159,160]. Molina et al. (2017) studied the onset of the changes in the gene expression of NOS isoforms and their protein levels, as well as NO level changes following striatum hypoxia/reoxygenation in aged rats. Rats were exposed to hypoxemic conditions for 20 min and then were exposed to reoxygenation for 0 h, 24 h, and 5 days, in which they were sacrificed immediately thereafter. NOS gene expression levels did not change at 0 h following hypoxia. After 24 h, eNOS gene expression and protein levels, along with nNOS protein levels, were increased in the aged rat striatum. On day 5 of the post-hypoxia, iNOS gene expression did not change significantly, while iNOS protein levels were increased. Moreover, eNOS gene expression was enhanced while nNOS gene expression was suppressed. The levels of NO did not change significantly in aged striatum after hypoxia and reoxygenation, despite the increased activities of NOS isoforms during this time span. Molina et al. (2017) suggested that aging might disrupt the production of NO by NOS via decreasing the availability of BH4 cofactor and the impairment of Ca^2+^ homeostasis. Moreover, reduced O_2_ levels following hypoxia amplified the decrease in the NO production by NOS. Furthermore, the decreased NO in the hypoxic striatum could disturb the NO vasodilatory response in the brain vessels, which could further increase the severity of the hypoxic damage to the brain. The increased activity of NOS following hypoxia in the aged striatum was thought to be the compensatory mechanism of the brain to combat the effects of hypoxia on reducing O_2_ levels in the brain. Due to the impaired production of NO by NOS in the aged brain, the O_2_-independent production of NO was increased by denitrating the nitrated tyrosine residues to produce NO. The O_2_-independent production of NO in the aged striatum was shown as a continuous decline in the nitrated proteins from 0 h until 5 days after hypoxia/reoxygenation [161]. Aging-related changes in gene expression and corticostriatal synaptic plasticity were examined by Chepkova et al. (2015) in the dorsal striatum of four mice groups, aged from young to old [162]. The findings revealed a substantial drop in transcripts encoding neuronal NOS and activation receptors (NR1 glutamate NMDA receptor subunit and D1 dopamine receptor) in aged mice (Figure 4).

### 3.3. Reproduction System Aging and NO

Estrogen can upregulate the endothelium-derived vasodilator factors, including NO and prostacyclin [163,164]. Following the estrogen drop after menopause, women might have accelerated vascular dysfunction, which means that estrogen can be a trigger for these processes [165,166]. The overlap of menopause with aging brought up the debate of whether the increased risk of cardiovascular disease in this period results from aging or menopause or both [167].

A vasoconstrictor prostanoid, thromboxane A2 (TXA2), activates the TXA2 receptor (TP) and has a primary role in the hemostasis of normal systemic vasculature [168,169]. Cyclooxygenase (COX) activation and elevated TP receptor expression are implicated in age-associated vascular diseases [170]. In humans, the production of COX-derived, endothelium-derived contractile factors is a feature of the aged blood vessels resulting in an earlier onset and speeding up of endothelial dysfunction [171].

NO can suppress the prostanoid production through the regulation of the COX activity and, conversely, the prostanoids, such as TXA2, can decrease the NOS expression and activity [172,173]. During the aging processes, endothelium-derived TXA2 increases vasoconstriction and endothelial dysfunction, which is amplified by decreased NO activity [174].

Vidal-Gómez et al. (2016) showed that in the absence of estrogen in young and aged ovariectomized mice, TP activation decreased NO bioavailability in the aorta by stimulating COX and the production of superoxide. The administration of estradiol restored the decreased NO bioavailability in the aorta due to TP activation. COX upregulation contributed partially to TXA2 contractile functions, which could be enhanced by aging and the absence of estrogen, while it could be suppressed due to the inhibition of COX by indomethacin. The production of superoxide, and therefore decreased NO bioavailability by endothelium-derived prostanoid TXA2 and TP activation, was also age- and estrogen-dependent, and could be prevented by suppressing COX. Interestingly, when estrogen was absent, COX inhibition resulted in enhanced bioavailability of NO [175].

Novensà et al. (2011) studied how aging alters the estrogen affects the NO development in an accelerated senescence mouse model [176]. Aged animals were similar to young animals in terms of DAF-2 or plasma nitrite/nitrate (NO_2_/NO_3_) in NO production. Estrogen treatment improved the production of NO in young animals by increasing eNOS expression but did not affect NO production in aged animals. Estrogen inhibited NADPH-oxidase and its production of superoxide anion (O_2_^−^). O_2_^−^ catabolizes the NO and decreases its levels. Therefore, estrogen increased NO levels by decreasing NO catabolism via O_2_^−^ through inhibiting the production of O_2_^−^ by NADPH-oxidase. In Novensà et al.’s (2011) study, estrogen reduced the development of O_2_^−^ in young female animals, while in the aged animals estrogen increased O_2_^−^ [176]. The latter was due to the upregulated estrogen receptor (ERb/ERa) expression ratios in the aged female animals and thus modulated the antioxidant effects of estrogen, enhancing its pro-oxidant activity. The capacity of E2 to cause the modulation of eNOS and a decline of O_2_^−^ was impaired by aging. Activation of ERα has been associated with augmented eNOS expression and NO formation, and its antioxidant mechanism of action. The recent studies on the ERα knockout mice demonstrated its importance in the regulation of eNOS. In the opposite, the knockout of ERβ caused the development of hypertension, regardless of its normal NO production. These outcomes suggested that ERα is the most important ER subtype accountable for NO formation by estrogen. Furthermore, augmented levels of ERβ could be related to cardiovascular risk, comprising coronary calcification and atherosclerosis [176].

**Figure 5:**
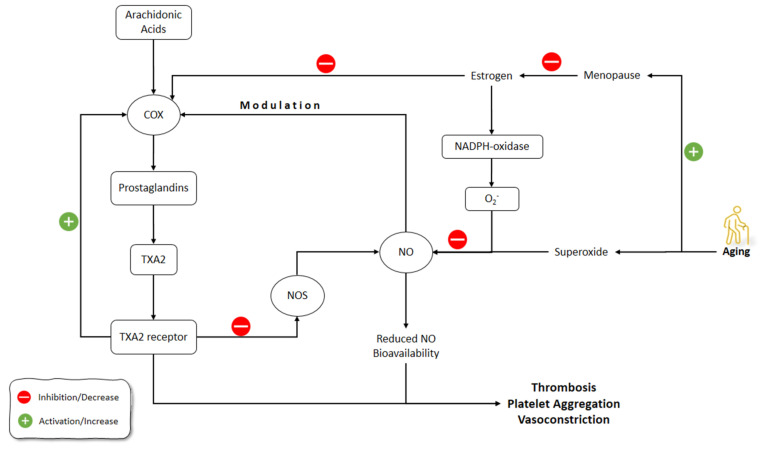
Effects of aging on estrogen and NO pathways involved in vasculature functions. NO: Nitric Oxide; NOS: Nitric Oxide Synthase; COX: Cyclooxygenase; TXA2: thromboxane A2.

Banerjee et al. (2012) showed that aromatase and ER-alpha were localized in Leydig cells of the testis and demonstrated a strong association between testicular aromatase and the level of circulating testosterone, implying that testicular steroidogenesis could be modulated by estrogen. The baseline level of NO during the reproductive activity cycle was shown in this study, although aged animals showed the correlation between decreased testicular activity and elevated serum NO. This research found that aromatase and NO levels were inversely associated. Furthermore, NO elevation downregulated steroidogenesis and germ cell survival. In summary, reduced estrogen enhances NO production in old age, which reduces testicular steroidogenesis and the apoptosis of germ cells [177] (Figure 5).

**Figure 6:**
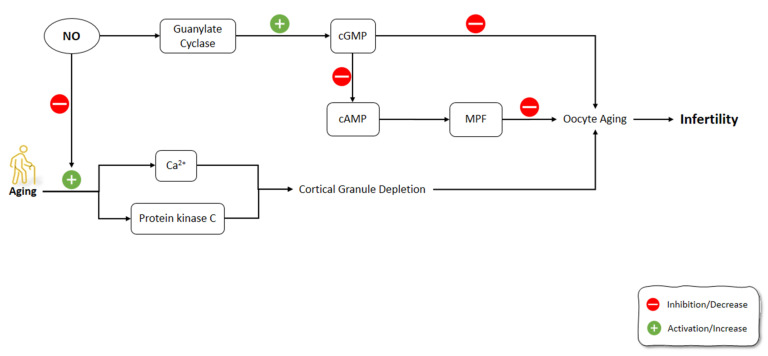
Effects of aging on the signaling pathways involved in oocytes aging including NO pathway. NO: Nitric Oxide; NOS: Nitric Oxide Synthase; cGMP: Cyclic guanosine monophosphate; cAMP: Cyclic adenosine monophosphate; MPF: M-phase-promoting factor.

One of the causes of reproductive failure is the oocyte’s postovulatory aging [178,179]. NO is a critical intracellular and intercellular messenger adjusting numerous physiological components in the processes of folliculogenesis, ovulation, and oviductal travel [180,181,182,183]. Goud et al. (2005) showed that NO exposure to young and old mice oocytes slowed the aging processes and increased the stability of microtubular spindle apparatus. The underlying mechanisms for these effects of NO were the activation of guanylate cyclase, which led to increased cGMP production [184]. Previous studies have shown that GMP alone can alleviate meiosis in rat and hamster oocytes. According to the outcomes of the aforementioned research, the production of cGMP occurred in the cumulus cells and was possibly transported into the oocyte via gap junctions to impede meiosis [185,186]. Similarly, specific PDEs are also triggered by cGMP and contribute to cAMP decrease [187]. This might activate the M-phase-promoting factor (MPF), a core regulator of M II phase arrest [188,189]. During the fertilization of oocytes, cortical granule depletion has been associated with aging-like changes such as elevated cytosolic Ca^2+^ and activated protein kinase C; however, these changes can occur in unfertilized oocytes due to aging [190,191]. In Goud et al.’s (2005) study, the mechanism of anti-aging effects of NO in oocytes might be related to the increase in Ca^2+^ levels or the suppression of protein kinase C activation [184] (Figure 6).

### 3.4. Skin Aging and NO

In aged people, skin loses its ability to increase blood flow during heat stress, which can lead to the increased occurrence of heat-related diseases and even to death [192,193,194,195,196]. The sympathetic nervous system regulates the blood flow to the skin via the adrenergic vasoconstrictor system and an active vasodilator system [197]. 

An increase in body core temperature releases tonic adrenergic vasoconstrictor which elevates the skin blood flow. After a threshold, as temperature rises, sweating and cutaneous active vasodilation (AVD) occur [197]. In young individuals, about 30% of AVD is controlled by NO [198]. In older populations, NO-dependent vasodilation of skin is compromised [199]. Holowatz et al. (2003) showed that during passive whole-body heating, NO-dependent AVD was 23% and 60% in young and old individuals, respectively. Thus, the NO-mediated pathways were related more to the total vasodilatory response of the aged subjects at high core temperatures. The authors suggested that an unknown transmitter(s) could mediate the AVD during passive whole-body heating in aged individuals [200]. 

In another study, Bruning et al. (2012) showed that eNOS is primarily responsible for mediating cutaneous NO-dependent vasodilation during the local heating and perfusion of the endothelium-dependent agonist ACh in middle-aged skin. In addition, the expression of NOS isoforms or downstream vasodilatory molecular targets did not differ in the biopsy studies [201,202].

### 3.5. Renal Aging and NO

Reckelhoff et al. (1994) showed that, during aging, NO production decreases, which could be explained by the lack of the endogenous substrate in aged rats, l-arginine [203].

Age-dependent kidney diseases are more common in male humans. NO signaling dysfunction can cause age-dependent kidney damage. Erdely et al. (2003) found that renal NOS decreased by aging in male but not female rats [204]. These data suggested that the difference of NOS activity and NO function might be a determining factor for the gender gap in the rate of renal functional decline in advancing age. According to the study, a noticeable sexual dimorphism demonstrated that the females were protected both by the presence of estrogens and the deficiency of harmful androgens. The total NO formation, the activity of NOS and its abundance were important in the aging male rat kidney. Although arginine production was conserved, which showed elevation in the circulating NOS inhibitor, ADMA, in the aging male rat, these effects improved deteriorations in NO production [204,205]. 

Doleželová et al. (2016) showed that renal NO and CYP450-epoxygenase products, which counteract the development of hypertension and protect the kidney, were decreased in a genetic animal model of spontaneous hypertension (fawn-hooded hypertensive or FHH rat). Renal NOS activity was higher in adult animals with established hypertension but was similar between prehypertensive FHH rats and age-matched non-hypertensive controls [206]. Reduced vasodilator response in isolated renal vessels from adult rats has been shown in in vitro studies [207,208]. 

Stadler et al. (2003) demonstrated that before the occurrence of histopathological injuries in diabetes, NO and peroxynitrite are increased in streptozotocin-induced diabetic rats. Moreover, the ability of increased NO levels to suppress the formation of ROS in vessels and kidney tissues highlighted the possible roles of NO and peroxynitrite in late diabetic complications [92]. 

Impaired angiogenesis is believed to cause the age-related nephropathy, along with the gradual weakening of renal microvasculature. Satoh et al. (2013) showed that human umbilical vein endothelial cells (HUVECs) treated with NOS inhibitor had higher expression and activity of cathepsin D. Cathepsin D produces angiostatin, a potent in vivo angiogenesis inhibitor. Aged rats treated with l-NAME, had higher cathepsin D and angiostatin activity. On the other hand, treatment with a long-lasting NO-releasing vasodilator diminished the production and activity of cathepsin D and angiostatin. It was suggested that one of the most important steps to generate angiostatin would be related to the contribution of the proteolytic cleavage of plasminogen. Besides, the inhibition of cyclooxygenase (COX) and MMP-2 activities were also related to the angiostatin-generating mechanisms in vivo. In summary, cathepsin D-induced production of angiotensin was increased in the aging rat kidney. Decreased NO output triggered activity of cathepsin D. Increased development of angiostatin in the aged rat kidney could be due to the capillary loss and interstitial damage. [209]. However, in the aforementioned study, no direct interplay was observed between the generation of angiostatin in tubulointerstitial damage related to the peritubular capillary loss in the aged kidney.

### 3.6. Thyroid Aging and NO 

The impairment of water reabsorption and improvements of aquaporin type 2 water channel (AQP2) were consistent with hypothyroid state and aging. AQP2 trafficking to the apical plasma membrane, in medullary duct collection cells, involves NO. Sarati et al. (2013) showed that the urine output and medullary NOS activity are correlated reversely in young and old rats, as the former was increased in the young and later was increased in the old rats. Elevated AQP2 in rats with hyperthyroidism was found in the plasma membrane of the young rats and at the cytosolic site in adult rats. These findings showed that medullary NO and AQP2 are implicated in maintaining water homeostasis [210]. Due to the role of the connection of NO-AQP2-hypothyroid damage in the regulation of water homeostasis, it was suggested that hypothyroidism may affect renal parameters in the aging processes.

Sarati et al. (2012) examined whether changes in NO development were age-related and contributed to hypothyroidism’s cardiovascular symptoms [211]. Thyroid hormones contributed to the modulation of cardiovascular NO activity as well as tissue-specific abundances of caveolin-1 and -3, regardless of age. Young animals with hyperthyroidism had reduced levels of three NOS isoforms, whereas the adult ones had increased caveolin-1 expression. Hypothyroidism was correlated with increased NOS activity in the ventricle and aorta, whereas atrial NOS activity was decreased in both young and adult animals. The increased NOS activity in the ventricle of young animals with hyperthyroidism was due to iNOS isoform, while in the aorta of both young and adult ones, it was related to eNOS and iNOS isoforms [211].

### 3.7. Erectile Dysfunction, Aging, and NO

The decrease of smooth muscle cells (SMCs) and collagen in the corpus cavernosum (CC) greatly contributes to erectile dysfunction (ED) during aging. One of the occurring phenomena during the aging process is tissue remodeling in the CC. The aforementioned tissue remodeling feasibly occurs by reason of phenotypic cellular alteration from SMCs to fibroblasts that latterly would cause an elevation in the content of collagen deposit [212]. According to anatomical localization, the corpora cavernosa are paired spongy cylinders that lay on the superior facet of the penis [213]. The accumulation of collagen is thought to be affected by ROS. The NO production by iNOS has been shown to suppress the ROS and thus decrease collagen accumulation [212]. To clearly describe the relationship between ROS formation and collagen accumulation, this process was described in Peyronie’s disease, which is accompanied by fibrosis and is characterized by an augmentation in collagen over the intracellular part. Fibrosis is related to the formation of profibrotic factors, including plasminogen activator inhibitor-1, transforming growth factor beta (TGF-β), and ROS throughout oxidative stress. This is accompanied by the stimulation of the iNOS, which plays a crucial part as an endogenous antifibrotic pathway in the case of exposure with profibrotic manners [214]. Inside the arterial media SMC, Ferrini et al. (2004) explored the possibility of related mechanisms that exist within aging and that share specific common physiological roles with the cavernosal SMC [215]. Male, brown Norwegian rats aged (22–24 months) were treated with an iNOS action inhibitor. Inhibition of iNOS activity was induced by l-*N*-(iminoethyl)-lysine acetate. Resistance arteries of the penis showed an increased SMC apoptosis, increased collagen amounts, and elevated ROS and iNOS levels. Administration of an iNOS action inhibitor worsened the SMC/collagen ratio and increased ROS levels. A prevalent etiology can be explained in the hypotheses of ED and arteriosclerosis in aging males, namely that the SMC’s production of iNOS is an effort to combat this fibrosis [215].

Ferrini et al. (2001) showed that, as aging advances, iNOS and peroxynitrite increases in the penis of old rats. According to the overproduction of NO by reason of the induction of iNOS, the apoptotic cell fate and aging were increased, but in order to elucidate this probable interaction, more studies should be conducted in the future. These changes were suggested to contribute to the increased apoptosis and proteolysis and higher collagen deposition as a result of iNOS and peroxynitrite induction in the old penises [216].

Garbán et al. (1995) analyzed adult, old, and senescent rats to determine whether aging can decrease the erectile response and possible correspondence with lower levels of NOS in the penis [217]. Aging-induced erectile dysfunction was independent of penile NOS deficiency but can be worsened by decreased NOS in very old rats. Old and senescent rats showed lower maximum intracavernosal pressure (MIP) in the Cavernosal nerve electric field stimulation (EFS) compared to adult rats. Old and senescent rats showed more decline in MIP in response to NOS inhibition compared with adult rats. Moreover, when NOS was not inhibited, old and senescent rats had a lower erectile response to intracavernosal papaverine (PDE inhibitor) or nitroglycerin (NO donor) compared to adult rats [217].

Haas et al. (1998) studied the aging-associated erectile dysfunctions, including endothelial dysfunction of cavernosum, upregulation of eNOS, and aberrant intracellular calcium flux in aged rabbits. It was found that relaxation of corporal tissue was significantly mitigated during the treatment of acetylcholine (ACh), an endothelium-dependent vasodilator. No difference was observed in corporal tissue relaxation due to the treatment of animals with an NO donor sodium, nitroprusside. The calcium ionophore increased the reduced vasorelaxation in the aged rabbits’ cavernosum and had no effect on the young rabbits’ cavernosum. These findings proposed that erectile dysfunction in the aging rabbit cavernosum was probably related to endothelial dysfunction and was characterized by eNOS upregulation and aberrant intracellular calcium fluxes. It was proposed that the defect in the ACh-NO pathway during the aging process was attributed to the level of NO synthesis, not its activity [218].

### 3.8. Muscle Aging and NO

During the mild to moderate-intensity exercises, the hyperemic and vasodilator response involves the NOS functions in humans [219,220,221]. Isolated arterioles of skeletal muscles from aged animals have shown slower endothelium-dependent vasodilation [222]. The reactive hyperemic response contributed to an impulsive intensification in muscle and skin blood flow after the discharge of arterial occlusion. This response was related to the modulation of the micro/macrovascular functions [223]. During exercise, Casey et al. (2015) analyzed, measured, and described the effect of aging on hyperemic and vasodilator kinetics [224]. In higher relative exercise intensities, aged individuals showed a higher attenuation in the amplitude of hyperemic and vasodilator. Moreover, blood flow and vasodilation of skeletal muscles in aged individuals were slower in rhythmic forearm exercises. Reduced bioavailability of NO was considered to be the underlying reason for these observations. Altogether, aging was associated with impaired blood supply control in the skeletal muscles during dynamic exercises [224].

During submaximal dynamic exercise, the age-induced decrease in the NO bioavailability leads to endothelial dysfunction that results in redistribution of blood flow from extremely oxidative (slow-twitch oxidative and fast-twitch oxidative glycolytic) to glycolytic (fast-twitch glycolytic) muscles [222,225]. 

It was found that the administration of l-NAME increased the mean arterial pressure, indicating that the NOS activity was attenuated. l-NAME administration also alleviated vascular conductance in the rats’ hindlimb muscles or muscle parts studied during high-speed treadmill exercise. The decreased vascular conductance correlated with the appraised summation of the percentage of slow-twitch oxidative and fast-twitch oxidative glycolytic sorts of fibers in every single muscle. The study results suggested that extremely oxidative muscles in young animals depend more on NO-mediated vasodilation than in mostly glycolytic muscles in the transition period from rest to exercise [226]. This was shown in a study by Hirai et al. (2011) in which aged animals had impaired NO regulation of regional hemodynamic during rest to submaximal whole-body exercise (total, inter-, and intramuscular hindlimb), especially in highly oxidative muscles [227]. 

Aging causes atrophy and progressive loss of function in muscles. The satellite cells of aged muscles are gradually refractory to stimulation that would reduce atrophy. Skeletal muscles have been shown to activate satellite cells by inducing NO release in vitro and in vivo. In vivo, NO produced by a muscle-specific isoform of NOS in fibers (NOS-Iµ) leads to activation of satellite cells. Besides, in vitro, the hepatocyte growth factor also upregulates satellite cells [228,229,230]. Skeletal muscle satellite cells are supposed to be responsible for the principal functions in muscle fiber maintenance, restoration, and remodeling. Satellite cells are considered to be the main site of the production of new myonuclei in postnatal skeletal muscle tissue. The term “satellite cell” was created due to its anatomical place. They are located between the sarcolemma and basal lamina of their related muscle fiber [231]. Leiter et al. (2010) showed that aging and subsequent changes in NOS activity leads to the gradual recalcitrance of satellite cells in adult mice muscles to mechanical stretches. These changes were alleviated by exogenous NO treatment [232].

Palomero et al. (2012) demonstrated that young single skeletal muscle fibers of mice had increased intracellular superoxide activity (elevated dihydroethidium oxidation) while having unchanged NO activity (reflect by intracellular DAF-FM or CM-DCFH oxidation) following passive stretch. The opposite was observed in the old mices’ skeletal muscle fibers [233].

Novella et al. (2013) showed that vascular response in aged mice aorta increased contraction by phenylephrine and decreased endothelium-dependent relaxation by acetylcholine. The reduced relaxing response of the aorta in aged mice was linked to the reduced NO production and eNOS expression [166]. 

Aging changes the phenotype of myeloid cells, which may affect the muscle structure. Wang et al. (2015) observed that aging led to increased anti-inflammatory M2a macrophages, collagen production, and subsequent muscle fibrosis. Usually, T-helper-2 cytokine interleukin (IL)-4 or IL-13 play a role in the activation of the macrophages of the M2 phenotype. Nevertheless, during skeletal muscle inflammation, the activation of the M2a phenotype requires IL-10, which is accompanied by an increased expression of CD163 and arginase. These changes were stopped in mice with muscle-specific nNOS transgene. The mechanism for these effects of nNOS transgene was linked to the prevention of the aging-induced increases of arginase-1 and related profibrotic signaling pathways [234]. 

The produced NO and superoxide in skeletal muscles react with each other to form peroxynitrite. This can affect the nearby reaction of superoxide dismutation to hydrogen peroxide. Pearson et al. (2015) showed that the aging-induced elevation of NO generation by eNOS increases the peroxynitrite and decreases the availability of superoxide in muscle mitochondria. The latter and increased nitration of muscle proteins by peroxynitrite can potentially contribute to age-related degenerative skeletal muscle changes [235]. During the contractile activity of skeletal muscles, NO and superoxide are produced. Superoxide and NO are the precursors for the formation of numerous secondary messenger species [236,237].

One of the deliberating muscle loss phenomena in aged individuals is sarcopenia. It has been shown that high levels of calpastatin, the endogenous inhibitor of calcium-dependent proteases (calpains), alleviates the severity of sarcopenia. In an animal model of sarcopenia, Samengo et al. (2012) showed that calpain in adult animals is inactivated by NO-mediated S-nitrosylation. The decreased nNOS and subsequent NO in aged animals’ low muscles alleviated the suppression of calpains and increased calpain-mediated proteolysis of myofibrils. Restoring the muscle-specific NO by nNOS transgene prevented sarcopenia in aged animals [238].

### 3.9. Sleep Problems, Aging and NO

NO induces endogenous sleep through adenosine in the cholinergic basal forebrain (BF) following prolonged wakefulness and sleep deprivation (SD) [239,240]. The inhibition of NO has been shown to impair sleep initiation while exogenous NO donors initiated it [239,240]. The mechanism through which NO induces sleep is impaired by normal aging [241]. NO-dependent adenosine release modulate sleep processes by inhibiting energy production. In vitro, NO donors promote glycolysis, augment adenosine, lactate, and pyruvate levels, and impede oxidative phosphorylation, causing a reduction in total energy assembly and a decrease in the ATP/ADP ratio [242,243].

Rytkönen et al. (2010) showed that aged rats exhibit decreased iNOS and NO in response to SD compared with young and middle-aged rats. Moreover, infusion of NO donor in the BF of aged rats did not change the sleep recovery response, which shows that sensitivity of the BF in aged animals is decreased towards NO [241].

Chronic SD affects about half of Alzheimer’s disease (AD) patients. The *N*-methyl-d-aspartate (NMDA)/NO pathway has been shown to affect the pathobiology of AD. Moreover, SD has been suggested to affect NMDA receptors which are involved in sleep-wake cycles [244,245]. Kristofikova et al. (2019) found that the expression of subunits of NMDA receptors (NR1, NR2A, and NR2B) and eNOS, as well as nNOS, were reduced in aged rats. Changes to the nNOS were correlated with NR1 and NR2B in both hemispheres of animals. Chronic SD led to eNOS dysfunction and NR2A increased activity, while the acute SD elevated iNOS in the right side of rats’ brains. Chronic SD and age both affected the NMDA prevalence and NOS functions in a similar manner in which AD does [246].

## 4. Interventional Modalities in NO Pathway during Aging

### 4.1. Recombinant Adenovirus

By insertion of the eNOS gene through a recombinant adenovirus into the corpora cavernosum of aged rats, Champion et al. (1999) observed an increase in the eNOS activity and cGMP levels in the tissue. Subsequently, the enhanced erectile response to exogenous nerve stimulation, acetylcholine, and zaprinast (an inhibitor of PDE5) has been reported as well [247,248].

### 4.2. NMDA Agonists and Inflammatory Stimuli

An aging-induced decrease in Na, K-ATPase activity in the CNS can increase ROS and possibly neurodegenerative processes [249]. The NMDA receptor/NO function can activate the Na, K-ATPase pathway, which can act as an activator of NF-κB. The latter can lead to cyclic GMP-PKG activation. Inflammatory stimuli such as LPS can increase excitatory glutamatergic transmission, especially at the NMDA receptor [250,251,252]. 

### 4.3. Intermittent Fasting

Vasconcelos et al. (2015) showed that intermittent fasting (IF) suppressed the age-induced changes in nNOS, cGMP, and thiobarbituric acid reactive substances (TBARS) through α2,3-Na, K-ATPase activity, 3-NT proteins, and iNOS gene expression. IF also reduced the neuroinflammation of LPS-induced NF-κB activation and iNOS in both young and aged animals [253]. 

## 5. Therapeutic Agents Affecting NO Signaling Pathway in Aging

### 5.1. Synthetic Agents

#### 5.1.1. PDE3 Inhibitors 

Cilostazol is a selective PDE3 inhibitor that is administered as an antiplatelet medication to treat intermittent claudication and ischemic hallmarks associated with chronic peripheral arterial obstruction and to avert the initiation of secondary cerebral ischemia. Besides, some other pharmacological impacts are attributed to cilostazol containing vasodilator and anti-dyslipidemic effects. Inhibition of superoxide anion formation, the proliferation of vascular SMCs, and restenosis were observed by treatment with cilostazol in animals and humans. Recently, cilostazol has been studied in numerous disorders that progress to endothelial dysfunction. Moreira et al. (2018) indicated that cilostazol (**a**) a PDE3 inhibitor could reduce oxidative stress, increase NO bioavailability, and allow endothelium-derived hyperpolarizing factor (EDHF)-type relaxation in the mesenteric resistance arteries of aged rats [254].

#### 5.1.2. PDE4 Inhibitors

According to research, PDE4 is extremely expressed in the cerebral cortex and hippocampus and has been revealed to play a part in the regulation of cognition, learning, and memory-associated signaling pathways. Shreds of evidence obtained from preclinical studies specified that inhibition of PDE4 might be a novel attitude for treating numerous brain-related disorders, such as depression, anxiety, schizophrenia, multiple sclerosis, and Alzheimer’s disease. Roflumilast has been approved by the United States Food and Drug Administration (FDA) for the treatment of peripheral inflammatory disease and severe chronic obstructive pulmonary disease (COPD). Santiago et al. (2018) found that roflumilast, (**b**) a PDE4 inhibitor, might have the potential to recover memory deficits in aged rats with chronic cerebral hypoperfusion. It was feasibly effective in the reduction of white matter injury, the increase of arginase-1 in primary microglia cells, and the decrease of iNOS [255]. 

#### 5.1.3. PDE5 Inhibitors

The concentration of NO is considerably augmented after sexual stimulation, which is attributed to the conversion of guanosine triphosphate (GTP) to cGMP. Subsequently, cGMP mitigates intracellular Ca^2+^ in the cavernosal smooth muscles, causing their relaxation. Oral PDE5 inhibitors containing sildenafil (**c**), tadalafil (**d**), vardenafil (**e**), and avanafil (**f**) can successfully decrease the metabolism of cGMP, finally leading to a positive erection. PDE5 inhibitors characterize a leading first-line oral therapeutic choice for men with ED [256].

Recently it was observed that chronic treatment of *mdx* mice (a murine model of Duchenne muscular dystrophy) with PDE5 inhibitors attenuated muscle fibrosis and increased in vitro force production, while acute treatment ameliorated muscle perfusion and elevated post-exercise activity levels. In the same way, acute treatment with PDE5 inhibitors in patients with muscular dystrophy ameliorates perfusion of active muscles throughout exercise procedure. Sheffield-Moore et al. (2013) showed that an increase in NO-cGMP signaling by sildenafil (**c**) could augment the synthesis of proteins, change nitrosylation and protein expression, and lessen fatigue in human skeletal muscle [257]. 

In aging-related cognitive decline studies, the improvement in learning and memory has been reportedly correlated to the modulation of NO-cGMP signal transduction following the activation of NMDA by sildenafil (**c**) in vivo [258,259].

#### 5.1.4. Hydroxymethylglutaryl-Coenzyme A (HMG-CoA) Reductase Inhibitors, “Statins”

ED is characterized by decreased NO-mediated corpus cavernosum relaxation and increased contractile activity by RhoA/Rho-kinase pathway [260,261,262]. The most frequent medications used in hyperlipidemic or diabetic patients are statins, which play a critical role in the mechanism of action, in which they impede the rate-limiting step in cholesterol biosynthesis. Statins decrease LDL and very-low-density lipoprotein (VLDL) levels, although they increase high-density lipoproteins (HDL) levels. Experimental evidence has demonstrated that statins can modulate endothelial function, including the increasing of NO generation, at doses that do not lower plasma lipids. Rosuvastatin (**g**) has been shown to correct NO function in nerves and vasculature in diabetic mice [263]. Statins can also increase NO bioavailability by enhancing both eNOS and nNOS expression [264,265]. Dalaklioglu et al. (2014) showed that pravastatin (**h**), a lipid-lowering drug with HMG-CoA reductase inhibitory actions, improved ACh- or EFS-induced corpus cavernosum relaxation as well as eNOS and nNOS expressions. Moreover, pravastatin restored the increased gp91phox, RhoA, and Rho kinase (ROCK2) expressions [266]. 

Han et al. (2012) reported that atorvastatin (**i**) might have promising antiaging potential in old rats with cardiac aging. Attenuation of left ventricle thickness, cardiomyocytes diameter, deposition of collagen, I/III collagen ratio, malondialdehyde (MDA), β-galactosidase, and augmentation of SOD, CAT, and NOS activities were observed by administration of atorvastatin. Repression in the expression of IL-1β, TNF-α, and MMP-9, and the intensification of the expression of PPAR-α/β/δ/γ were perceived as well [267]. 

#### 5.1.5. β-Blockers

Recent studies have shown that second- and third-generations of β-blockers have an impact on vasodilatation via endothelial β2-adrenergic receptor-mediated NO generation, anti-oxidative properties, and/or ATP efflux with the subsequent motivation of P2Y-purinoceptor-mediated NO production. Metoprolol has been revealed to be efficient in decreasing the risk of morbidity and mortality associated with cardiovascular diseases, e.g., heart failure, an age-related disease described by endothelial dysfunction. In a study by Funovic et al. (2008), administration of metoprolol (**j**), a β1-selective blocker in aged rats, reversed eNOS uncoupling, augmented the rate of formation and NO availability, and improved a whole ratio of NO/ONOO according to the endothelial function [268].

#### 5.1.6. 5-Hydroxytryptamine Subtype 3 (5-HT3) Receptor Antagonists

5-Hydroxytryptamine subtype 3 (5-HT3) receptor antagonists containing tropisetron are broadly administered to combat the induction of emesis post-surgical operation and after chemotherapy. Several studies have revealed other potential uses for 5-HT3 inhibitors. Moreover, a number of studies have found the neuroprotective potential of the 5-HT3 receptor antagonists in vitro and in vivo. Improvement in ROS production and apoptosis was reported after the administration of 5-HT3 receptor antagonists to amyloid-β-treated rat cortical neurons. Furthermore, in vivo research studies have demonstrated their outstanding immunomodulatory impacts in CNS. The anti-inflammatory, antioxidant, and anti-apoptotic properties of tropisetron were proved in various in vitro and in vivo studies. Mirshafa et al. (2020) investigated the effects of tropisetron (**k**) on D-galactose brain aging mice. It was reported that tropisetron significantly raised the gene expression of SIRT1, reduced markers related to oxidative stress and mitochondrial dysfunction, alleviated NO, TNF-α, and IL-6, and suppressed apoptosis [269].

#### 5.1.7. PPAR-γ Agonist

Recent studies have revealed that aging may be related to reduced PPAR-γ expression in old humans. Pioglitazone is a recognized PPAR-γ agonist that is administered in the treatment of type 2 diabetes. Wang et al. (2014) discovered that pretreatment of aged rats’ cerebral arteries with pioglitazone (**l**) improved ROS generation, eNOS phosphorylation, and NO levels related to the improvement of endothelium-dependent relaxation. Administration of pioglitazone also reinstated the expression of PPAR-γ and augmented the levels of mitochondrial uncoupling protein 2 (UCP-2) in aged rat cerebral arteries [270]. 

#### 5.1.8. eNOS Cofactors

**Figure 7:**
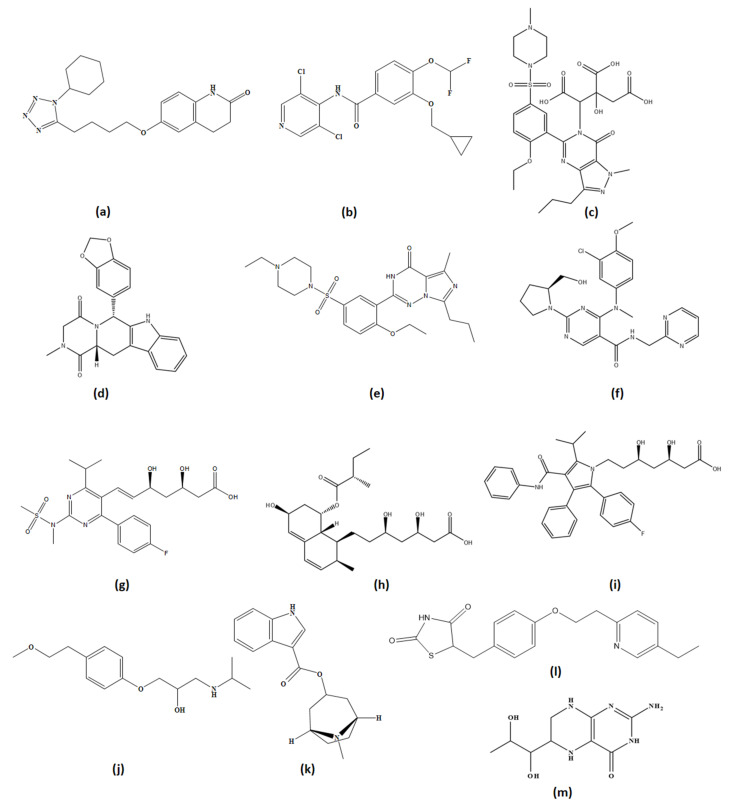
Chemical structures of synthetic therapeutic agents affecting NO signaling. See Table 1 for explanations of (**a**–**m**).

BH_4_ is an allosteric factor that is present in the coupling of the oxidase and reductase domains of eNOS. According to recent experimental evidence, BH_4_ deficiency causes an alleviation in NO production and an increase in ROS formation. In animals and individuals of progressed age, eNOS dysfunction together with a BH_4_ deficiency leads to ROS generation and decreased NO bioavailability. Besides, it has been revealed that the supplementation of BH_4_ or folic acid ameliorates endothelial function in smokers and patients with diabetes, hypercholesterolemia, hypertension, and chronic heart failure. Higashi et al. (2006) investigated the response of forearm blood flow to either endothelium-dependent vasodilator or endothelium-independent vasodilator with or without BH_4_ (**m**) treatment in healthy men. BH_4_ increased the relaxation of forearm vessels subjected to endothelium-dependent vasodilator but not the endothelium-independent vasodilator. The enhancing vasorelaxation of BH_4_ was abolished following treatment with an NOS inhibitor [271] (Figure 7) (Table 1). 

### 5.2. Natural Agents

#### 5.2.1. Polyphenols

Resveratrol is a plant polyphenol isolated from various foods such as nuts, grapes, and chocolate. Resveratrol has shown various efficient potentials in preclinical and clinical assays including cardioprotective effects, anti-diabetes, antioxidant and anti-inflammatory properties. Zhao et al. (2018) indicated that resveratrol (**a′**) led to the activation of SIRT1, which motivated the release of bone morphogenic protein 2 via eNOS in eNOS−/− mice connected to skeletal aging [272]. In a research work performed by Ota et al. (2013), it was observed that trans-resveratrol isolated from *Gnetum gnemon* augmented expression of eNOS and SIRT1 in the induction of endothelial dysfunction by H_2_O_2_ in aged HUVECs [273]. 

Honokiol, a small-molecule polyphenol extracted from *Magnolia officinalis*, showed various pharmacological activities, such as anti-thrombotic, anti-inflammatory, and anti-cancer properties. Liu et al. (2020) showed that honokiol (**b′**) suppressed atherosclerotic plaque formation in ApoE−/− mice fed with a Western-type diet. The possible mechanisms in which honokiol inhibited the atherosclerosis process were through the suppression of NF-κB pathway and NO production, which resulted in antioxidative and anti-inflammatory actions [274]. 

Daily intake of cacao-derived products has a reverse relationship with the incidence of cardiovascular diseases such as heart failure, myocardial infarction, and stroke. Catechins, natural flavanols, have presented a vast range of biological effects including antioxidant, anti-inflammatory, anti-cancer, neuroprotective, and cardioprotective impacts. In a study by Ramirez-Sanchez et al. (2018), cacao products rich in (−)-epicatechin (**c′**) recovered the decreased NO levels in bovine coronary artery endothelial cells (BCAECs) and in the aortas of aged rats. Furthermore, (−)-epicatechin suppressed the acetylation of eNOS by increasing the protein–protein interaction of eNOS with sirtuin-1 [275,276]. Garate-Carrillo et al. (2020) investigated the role of epicatechin, a flavonoid, on aged BCAECs and aged rats. This treatment led to a reduction in the activity of arginase and oxidative stress, restored the eNOS monomer/dimer ratio and NO generation, and ameliorated vascular function [277]. 

Baicalein is a flavone compound (**d′**) isolated from the root of *Scutellaria baicalensis*. Baicalein has exhibited several beneficial applications, including its anti-tumor, anti-apoptosis, anti-fibrosis, and anti-inflammatory effects. Zhang et al. (2014) revealed the anti-inflammatory activities of baicalein (**d′**) in human osteoarthritic chondrocytes. Baicalein exerted its anti-inflammatory potential in connection to anti-apoptotic and anti-catabolic effects. Alleviation of NO production and caspase cascade as downstream molecules were related to its anti-apoptotic actions. However, the anti-catabolic mechanisms retrieval in the deposition of glycosaminoglycan and type II collagen were observed. Besides, mitigation of matrix metalloproteinases (MMP)-3 and -13 induced by TNF-α and IL-1β were displayed following treatment with baicalein [278].

Li et al. (2021) showed that the administration of icariin (**e′**), a flavonol glycoside in aged rats, could enhance learning skills and motor coordination. Additionally, the attenuation of components of oxidative stress in numerous biofluids and organelles, and a decrease of pro-inflammatory cytokines and iNOS were exhibited, which also showed a correlation with the regulation of gut microbiota in the old animals [279]. 

#### 5.2.2. Curcuminoids

Sun et al. (2015) showed that pretreatment of H2O2-treated HUVECs with curcumin (**f′**) mitigated oxidative stress and apoptosis, and partially restored eNOS phosphorylation, NO bioavailability, and SIRT1 expression [280]. In a study directed by Yu et al. (2013), curcumin improved memory deficits in aged mice, partially by modulating the nNOS activity in the prefrontal cortex, hippocampus, and amygdala [281]. 

#### 5.2.3. Chalcone Derivatives

Luo et al. (2016) revealed the putative therapeutic potential of 2′-hydroxy-4,3′,4′,6′-tetramethoxychalcone (**g′**) in the response to the induction of inflammation in BV2 microglial cells by LPS. The aforementioned chalcone derivative, which was isolated from *Chloranthus henryi*, suppressed the expression of iNOS and COX-2, the production of ROS and NO, the secretion of IL-1β, TNF-α, and IL-6, the phosphorylation of c-Jun N-terminal kinase (JNK) 1/2 and the route of its translocation into the nucleus, and the stimulation of activator protein-1 [282].

#### 5.2.4. Sphingolipids 

Lee et al. (2013) elucidated that glucosylceramide (**h′**), a plant-derived sphingolipid, could enhance the diminishing memory in aged mice. In the aforementioned study, the disturbance was associated with LPS. Glucosylceramide attenuated iNOS, COX-2, IL-1β, and TNF-α at mRNA levels [283]. 

#### 5.2.5. Phytocannabinoids 

Santiago et al. (2019) found that the administration of cannabidiol (**i′**), a phytocannabinoid existent in the *Cannabis sativa* plant in streptozotocin-treated middle-aged rats, promoted memory performance and abridged levels of inflammatory markers in the hippocampus, including iNOS, glial fibrillary acidic protein, ionized calcium-binding adapter molecule 1, and arginase 1. It also mitigated brain-derived neurotrophic factor (BDNF) stimulation following chronic cerebral hypoperfusion in diabetic rats [284]. 

#### 5.2.6. Pyranocoumarins

Decursin and its isomers have shown antiangiogenic, anti-inflammatory, anti-cancer, and anti-amnesic activities. He et al. (2021) revealed the anti-inflammatory properties attributed to decursin (**j′**), isolated from *Angelica gigas* in osteoarthritis in mice and in in vitro experiments. Alleviation in the levels of prostaglandin E2 (PGE2), IL-6, TNF-α, COX-2, NO, and iNOS have been reported, as well as the reduction of MMPs and ADAMTS and the regulation of phosphatidylinositol-3-kinase (PI3K)/Akt/NF-κB axis [285]. 

#### 5.2.7. Ginsenosides

Over 130 ginsenosides were isolated from ginseng. Among these ginsenosides, compound K (**k′**) has shown numerous biological and pharmacological activities, comprising anti-cancer, anti-tumor, anti-inflammatory, antioxidant, and anti-diabetic effects. Kang et al. (2016) studied the protective role of ginsenoside compound K (**k′**) as a minor phytoconstituent of *Panax ginseng* Meyer in aging encouraged osteoarthritis. Ginsenoside compound K (**k′**) suppressed generation of NO and ROS in H_2_O_2_-motivated mouse osteoblastic cells. Additionally, an increase in the levels of osteogenic markers including alkaline phosphatase activity and type I collagen was observed, whereas this compound decreased the expression of IkBα kinase and IL-1β [286].

#### 5.2.8. Triterpenoid Saponins

Astragaloside has demonstrated anti-inflammatory, immunomodulatory, anti-oxidative, and anti-cancer activities. In a study conducted by Li et al. (2019), anti-inflammatory properties of astragaloside IV (**l′**), the natural active constituent of *Astragalus membranaceus*, were exhibited in chondrocytes of aged patients and a mouse model of osteoarthritis. Astragaloside IV affected the suppression of various pathways entailed in the production of inflammatory markers induced by IL-1β. Regarding this influence, astragaloside IV inhibited the production of IL-6, TNF-α, NO, PGE2, the signaling of NF-κB, and the expression of MMP-13 and a disintegrin and metalloproteinase with thrombospondin motifs (ADAMTS)-5 [287]. 

#### 5.2.9. Monoterpenes

Essential oils have a complex chemical composition that possess various secondary metabolites. Terpenes, especially monoterpenes, have been recognized as the main components of essential oils. Monoterpenes of essential oils show potent antioxidant and anti-inflammatory activities. Karthikeyan et al. (2019) reported that α-pinene (**m′**) pretreatment improved oxidative damage and lipid peroxidation induced by UVA irradiation in the mouse skin. Moreover, the inhibition of pro-angiogenic factors (iNOS and vascular endothelial growth factor (VEGF)), inflammatory markers (TNF-α IL-6 and COX-2), and NF-κB p65, as well as the expression of apoptotic markers (Bcl-2-associated X protein (Bax), Bcl-2, and caspase-3 and -9) in the mouse skin, have been reported. Alpha-pinene could effectively suppress MMP-2, -9, and -13 expression in the mouse skin regarding photoaging [288]. 

Thymoquinone has demonstrated several biological indications in research studies including antioxidant, anti-inflammatory, antihyperglycemic, immunomodulatory, hepatoprotective, gastroprotective, nephroprotective, neuroprotective, and anti-tumor effects. It also exhibits activities. Idris-Khodja and Schini-Kerth (2012) reported the potential of thymoquinone (**n′**), a monoterpene compound in the amelioration of endothelial function in aging. An intake of thymoquinone in middle-aged rats led to the normalization of expression in eNOS and Ca^2+^-activated K^+^ channels. Moreover, components of the angiotensin system and oxidative stress were returned to normal by this phytoconstituent of *Nigella sativa* [289,290]. 

#### 5.2.10. Carotenoids

Vitamin A (β-carotene) has been efficaciously applied contrary to oxidative stress produced by ultraviolet (UV) radiation and consequently avoids melanoma and processes of skin aging. Valacchi et al. (2009) observed that β-carotene (**o′**) could downregulate the stimulation of MIP2, TNFα, iNOS, and HO-1 in ozone-induced aging in murine skin [291].

El-Baz et al. (2019) found that zeaxanthin (**p′**) isolated from *Dunaliella salina* microalgae could improve aging-related cardiac dysfunctions in D-galactose-treated rats. Administration of zeaxanthin caused a decrease in IL-6 and iNOS and an increase in glucose transporter-4 and SOD. Additionally, the activation of retinoid receptor-α in cardiac tissue has been reported as well [292]. 

In a research study directed by Choi et al. (2008), astaxanthin (**q′**) revealed promising protective properties in the LPS model of microglial cell stimulation. Astaxanthin potentially blocked COX-2 and iNOS, which were associated with feasible neuroinflammation during aging [293]. 

#### 5.2.11. Alkaloids

Higenamine is a chemical substance isolated from numerous plants. It has various beneficial activities including analgesic, anti-tumor, antioxidant, and anti-inflammatory effects. Bai et al. (2019) studied the anti-inflammatory activity of higenamine (**r′**) in aging-related intervertebral disc degeneration. Evaluation of the anti-inflammatory mechanisms of this natural alkaloid was assessed in human nucleus pulposus cells (NPCs) stimulated by IL-1β. Higenamine repressed NF-κB signaling pathway and alleviated levels of iNOS, NO, PGE2, COX-2, TNF-α, IL-6, MMP-3 and -13, and ADAMTS-4 and -5 [294]. 

#### 5.2.12. Miscellaneous

Tang et al. (2020) reported that arctigenin (**s′**), a phenylpropanoid dibenzylbutyrolactone lignan isolated from seeds of *Arctium lappa*, could act as a nutritive phyto-oestrogen and decrease PGE2, COX-2, iNOS, NO, IL-6, and TNF-α in IL-1β-stimulated human chondrocytes obtained from aged people with osteoarthritis. The inhibition of PI3K/Akt and NF-κB pathways were other defensive contributors in the anti-inflammatory process attributed to arctigenin [295].

Alpha-lipoic acid is an organosulfur compound (**t′**) that has been used in order to prevent/treat impediments connected to diabetes, e.g., protein glycation, irregular glucose consumption, polyneuropathy, and cataracts. Sena et al. (2008) has indicated that the treatment of aged and high-fat, diet-fed diabetic rats with α-lipoic acid (**t′**) restored endothelial function and ameliorated the oxidative damages. These promising therapeutic actions were partially attributed to eNOS recoupling and augmentation of NO bioavailability [296]. 

Dioscin has shown great antioxidant and anti-inflammatory activities. The role of dioscin, a natural steroid saponin isolated from *Dioscoreae rhizoma* on the aging brain in H2O2-treated PC12 cells and d-galactose-induced aging rats was studied by Qi et al. (2019). Dioscin (**u′**) regulated mitogen-activated protein kinase (MAPK or MAP kinase) and nuclear erythroid 2-related factor 2 (Nrf2)/antioxidant response element (ARE) pathway and adjusted content of oxidative damage and inflammatory markers. Moreover, an amelioration of memory and spatial learning and the reduction in levels of NOS in brain tissue were detected [297,298].

Ergostatrien-3β-ol (EK100) was isolated from the entire submerged broth of the mushroom *Antrodia camphorate*. Various biological indications have been considered for this herb and its components. Hence, it has antioxidant, anti-inflammatory, vasodilatory, and hepatoprotective effects. EK100 has anti-diabetic, anti-dyslipidemia, and anti-inflammatory effects, based on the data obtained during the preclinical experiments. In a study of UVB-induced erythema, wrinkle creation, and the epidermal thickness in hairless mice skin, the protective effects of ergostatrien-3β-ol (**v′**) were observed by Kuo et al. (2016). Study outcomes showed that application of this compound could topically inhibit the expression of IL-6, MMP-1, iNOS, and NF-κB. MMP-1 inhibition and anti-inflammatory responses were responsible for the suppression of collagen degradation in the skin. Moreover, ergostatrien-3β-ol induced protection against photoaging through the decrease of the transepidermal water loss [299]. 

In a study by Saleh et al. (2019), sulforaphane (**w′**), an organic isothiocyanate that is present in cruciferous vegetables, showed protective activities in a model of liver aging induced by d-galactose in rats. Treatment with sulforaphane enhanced liver biomarkers. Moreover, sulforaphane modified oxidative stress, decreased NO, protein carbonyl, TNF-α, and TGF-β, and prevented dysregulation of hepatic Nrf2/Keap1/HO-1 in aged rats [300].

Wang, et al. (2013) reported that forsythiaside (**x′**), a phenylethanoside isolated from *Forsythia suspense*, could efficiently improve memory performance, alleviate the levels of IL-1β, NO, MDA and norepinephrine (NE) levels, and upsurge activities of total superoxide dismutase (T-SOD), GPx and glutamate (GLU), and ACh levels in brain homogenates of aged SAMP8 mice [301].

Melatonin, an endogenously synthesized indolamine, is the major hormone of the pineal gland, and can act as both an antioxidant and a regulator of mitochondrial bioenergetic function, which mostly represents neurodegenerative diseases and aging-related disorders. In a rat study of sepsis, melatonin (**y′**) could efficiently reduce the aging-promoted iNOS expression [302,303].

Omega-3 polyunsaturated fatty acids (PUFAs) containing eicosapentaenoic acid (EPA) and docosahexaenoic acid (DHA) have displayed cardioprotective effects by motivating the endothelial production of NO. Farooq et al. (2020) found that the treatment of old rats with omega-3 preparation EPA: DHA 6:1 restored endothelium-dependent NO-intervened relaxation, made the angiotensin-converting enzyme (ACE)/angiotensin type 1 receptor (AT1R)/NADPH oxidase into normal levels, and attenuated ROS production in the mesenteric artery [304]. 

#### 5.2.13. Probiotics

Qian et al. (2018) reported that *Lactobacillus plantarum* LP-CQPC11 could work as a probiotic effective in the antagonization of oxidation and aging induced by d-galactose in mice. LP-CQPC11 increased the levels of SOD, GSH-Px, and GSH, while it diminished the levels of NO and MDA in the serum, liver, and spleen of the aforementioned animals. Furthermore, LP-CQPC11 successfully upregulated the expression of nNOS, eNOS, Mn-SOD, Cu/Zn-SOD, CAT, HO-1, y-glutamylcysteine synthetase, Nrf2, and NAD(P)H dehydrogenase [quinone] 1. LP-CQPC11 effectively upregulated SOD1, SOD2, CAT, GSH1, and GSH2 protein expression in mouse liver and spleen tissues as well [305].

#### 5.2.14. Amino-Acids 

**Figure 8:**
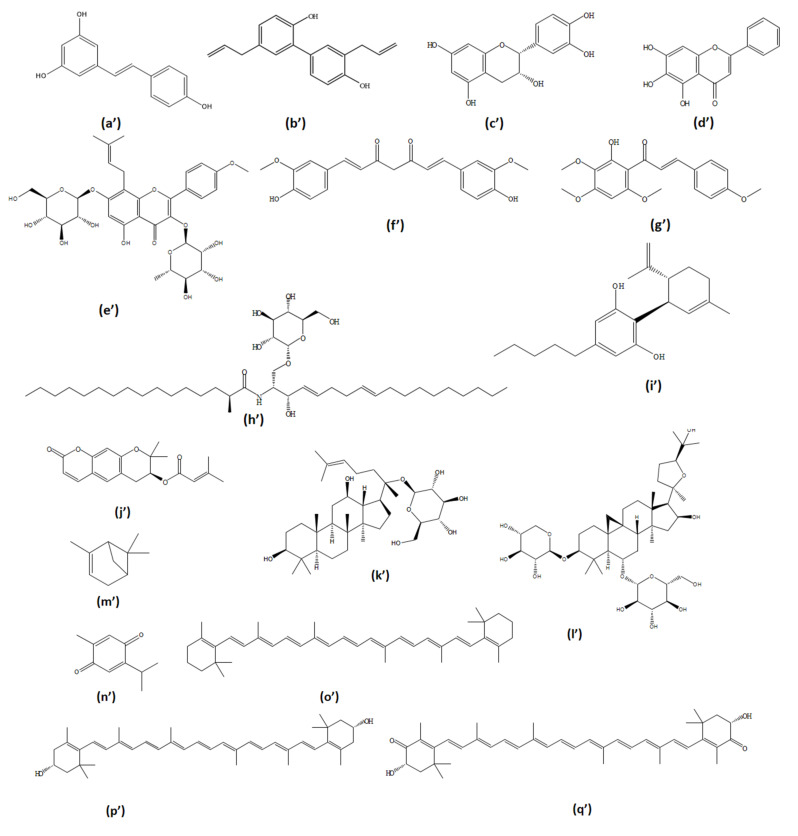

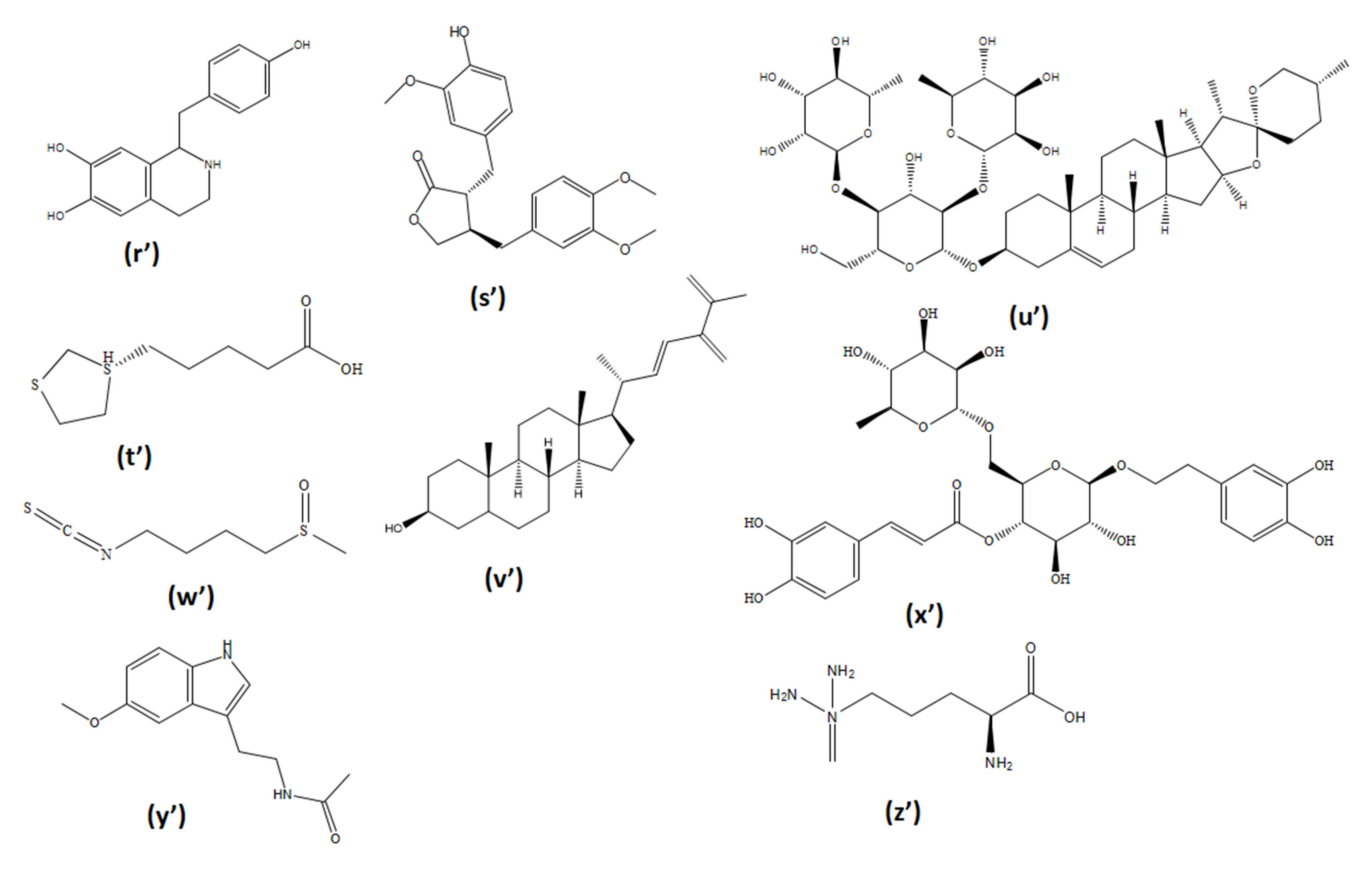
Chemical structures of natural therapeutic agents affecting NO signaling. See Table 2 for explanations of (**a′**–**z′**).

Zhong et al. (2010) showed that l-arginine (**z′**) could play a role in reversing senescent changes in HUVECs exposed to high levels of glucose. l-arginine suppressed the alleviation of eNOS and Akt activities [306] (Figure 8) (Table 2).

## 6. Conclusions

NO production and hemostasis play a main role in human health and disease. Furthermore, the physiological and pathophysiological effects of this molecule are very important in the regulation of aging processes. However, the complex activity of NO is related to several factors including cell type, NO production and bioavailability, as well as the type of enzymatic synthase and its reaction with target proteins. NO modulates various cellular processes during aging, inflammation, and age-related diseases, including cardiovascular, neurological, reproductive, skin, renal, thyroid, muscle, and sleep disorders. Several chemical and natural agents can increase NO bioavailability by enhancing both eNOS and nNOS expression, inhibiting iNOS, and increasing the protein–protein interaction of eNOS with sirtuin-1, leading to amelioration of aging-related diseases. However, due to the complex activity of NO in the pathogenic processes of diseases, several targets of NO should be considered, rather than a single target, to be able to identify the growing network of NO signaling processes in the body.

## Figures and Tables

**Table 1:** Synthetic therapeutic agents affecting NO signaling in the aging process.

Class of Compound	Synthetic Agent	Study Characteristics	Chemical Structure	Aging-Related Condition	Outcomes	Direct/Indirect Modulation of NO and NOS	Ref.
PDE3 inhibitor	Cilostazol	Aged rats	**a**	Endothelial dysfunction-type relaxation in mesenteric resistance arteries	↓Oxidative stress, ↑NO bioavailability and EDHF-type relaxation	↑NO bioavailability → ↑total and phosphorylated Akt → ↑eNOS phosphorylation (Akt–eNOS–NO pathway)	[254]
PDE4 inhibitor	Roflumilast	Aged rats	**b**	Memory deficits with chronic cerebral hypoperfusion	↓White matter injury, ↑arginase-1 in primary microglia cells, ↓iNOS	↓iNOS as an inflammatory marker	[255]
PDE5 inhibitor	Sildenafil, tadalafil, vardenafil, and avanafil	Clinical trials (aged men)	**c**–**f**	Erectile dysfunction	↓Metabolism of cGMP → positive erection	Modulation of NO-cGMP signaling	[256]
PDE5 inhibitor	Sildenafil	Clinical study	**c**	Fatigue in human skeletal muscle	↑NO-cGMP signaling → ↑synthesis of proteins, changes in nitrosylation, and protein expression	Modulation of NO-cGMP signaling	[257]
PDE5 inhibitor	Sildenafil	Rats with NOS inhibition	**c**	Aging-related cognitive declines	↑Learning and memory, modulation of NO-cGMP signal transduction, activation of NMDA	Modulation of NO-cGMP signaling	[258,259]
HMG-CoA reductase inhibitor	Rosuvastatin	Diabetic mice	**g**	Diabetes	Corrected NO function in nerve and vasculature	Regulated NO-ACh pathway	[263]
HMG-CoA reductase inhibitor	Pravastatin	Aged rats	**h**	Erectile dysfunction	Improved ACh- or EFS-induced corpus cavernosum relaxation, ameliorated eNOS and nNOS expressions, restored the increased gp91phox and RhoA/Rho-kinase expressions	Downregulated NADPH oxidase/Rho kinase, ↑eNOS/nNOS levels	[266]
HMG-CoA reductase inhibitor	Atorvastatin	Old rats with	**i**	Cardiac aging	↓Left ventricle thickness, cardiomyocytes diameter, deposition of collagen, I/III collagen ratio, MDA, β-galactosidase, ↑SOD, CAT, and NOS activities, repression in expression IL-1β, TNF-α and MMP-9, ↑expression of PPAR-α/β/δ/γ	Upregulated PPARs, ↑NOS activities	[267]
β1-selective blocker	Metoprolol	Aged rats	**j**	Endothelial dysfunction	Reversed eNOS uncoupling, ↑rate of NO production, NO availability, improved NO/ONOO	↑NO, ↓ONOO^−^, and restoring NO/ONOO^−^ balance	[268]
5-HT3 antagonist	Tropisetron	d-galactose-induced brain aging in mice	**k**	Brain aging	↑SIRT1 gene expression of, ↓markers related to oxidative stress and mitochondrial dysfunction, ↓NO, TNF-α and IL-6, and suppressed apoptosis	SIRT1 signaling	[269]
PPAR-γ agonist	Pioglitazone	Aged rats	**l**	Cerebral arteries aging	Improved ROS generation, eNOS phosphorylation, and NO levels, restored the expression of PPAR-γ, ↑levels of mitochondrial UCP-2	PPAR-γ targeting	[270]
eNOS cofactor	BH_4_	Clinical study	**m**	Vasorelaxation	↑Relaxation of forearm vessels subjected to endothelium-dependent vasodilator but not the endothelium-independent vasodilator	↑BH_4_ → stimulated eNOS and ↑NO production	[271]

**Table 2:** Natural therapeutic agents affecting NO signaling in the aging process.

Class of Compound	Natural Agent	Study Characteristics	Chemical Structure	Aging-Related Condition	Outcomes	Direct/Indirect Modulation of NO and NOS	Ref.
Polyphenols	Resveratrol	eNOS−/− mice	**a′**	Skeletal aging	Activated SIRT1 → motivated the release of bone morphogenic protein 2 via eNOS	SIRT1 signaling	[272]
Polyphenols	Trans-resveratrol	Aged HUVECs	**a′**	Endothelial dysfunction	↑eNOS and SIRT1 expressions	SIRT1 signaling	[273]
Polyphenols	Honokiol	ApoE−/− mouse fed with Western-type diet	**b′**	Atherosclerosis	Suppressed the atherosclerotic plaque formation, suppressed NF-κB pathway and NO production	Inhibited NO and iNOS expression	[274]
Polyphenols	Epicatechin	Aged rats	**c′**	Endothelial cell aging	Recovered the decreased NO levels in BCAECs and aortas, suppressed the acetylation of eNOS by increasing the protein-protein interaction of eNOS with SIRT1	SIRT1 signaling → suppressed eNOS acetylation	[275,276]
Polyphenols	Epicatechin	Aged rats	**c′**	Endothelial cell aging	↓Arginase activity and oxidative stress, restored the eNOS monomer/dimer ratio and NO generation, and improved vascular function	Modulated arginase and eNOS protein levels and activity	[277]
Polyphenols	Baicalein	Human osteoarthritic chondrocytes	**d′**	Osteoarthritis	↓NO production and caspase cascade, the anti-catabolic mechanisms recovery in the deposition of glycosaminoglycan and type II collagen, ↓MMP-3 and MMP-13	Mediated apoptosis via decrease of NO production	[278]
Polyphenols	Icariin	Aged rats	**e′**	Motor coordination and learning skills	↓Oxidative stress, ↓pro-inflammatory cytokines and iNOS, a correlation with the regulation of gut microbiota	↓iNOS, upregulated aging related signaling pathways e.g., SIRTs	[279]
Curcuminoids	Curcumin	HUVECs	**f′**	Endothelial cell aging	↓Oxidative stress and apoptosis, and partially restored eNOS phosphorylation, NO bioavailability, and SIRT1 expression	SIRT1 signaling	[280]
Curcuminoids	Curcumin	Aged mice	**f′**	Memory deficits	Improved memory deficits partially by modulating the nNOS activity in the prefrontal cortex, hippocampus, and amygdala	Activated nNOS/NO pathway	[281]
Chalcone derivatives	2′-hydroxy-4,3′,4′,6′-tetramethoxychalcone	BV2 microglial cell	**g′**	Neurodegeneration	Suppressed the expression of iNOS and COX-2, production of ROS and NO, secretion of IL-1β, TNF-α, and IL-6, phosphorylation of JNK 1/2, nuclear translocation, and stimulation of activator protein-1	Inhibited NO and iNOS as inflammatory markers	[282]
Sphingolipids	Glucosylceramide	Aged mice	**h′**	Memory deficits	↓mRNA levels of iNOS, COX-2, IL-1β, and TNF-α	Inhibited iNOS as inflammatory marker	[283]
Phytocannabinoids	Cannabidiol	Streptozotocin-treated middle-aged rats	**i′**	Memory deficits	↑Memory performance, ↓levels of inflammatory markers in the hippocampus including iNOS, glial fibrillary acidic protein, ionized Ca^2+^-binding adapter molecule 1, and arginase 1, ↓BDNF	↓iNOS, ionized Ca^2+^-binding adapter molecule 1, and arginase 1 as inflammatory markers	[284]
Pyranocoumarins	Decursin	Aged mice, in vitro	**j′**	Osteoarthritis	↓Levels of PGE2, IL-6, TNF-α, COX-2, NO, and iNOS, ↓MMPs and ADAMTS, regulated PI3K/Akt/NF-κB axis	Mediated NO and iNOS as inflammatory markers	[285]
Ginsenosides	Ginsenoside compound K	H_2_O_2_-motivated mouse osteoblastic cells	**k′**	Osteoarthritis	Suppressed generation of NO and ROS, ↑levels of osteogenic markers including alkaline phosphatase activity and type I collagen, ↓expression of IkBα kinase and IL-1β	Suppressed NO production	[286]
Triterpenoid saponins	Astragaloside IV	Chondrocytes of aged patients and a mouse model of osteoarthritis	**l′**	Osteoarthritis	Inhibited the production of IL-6, TNF-α, NO, PGE2, signaling of NF-κB, and expression of MMP-13 and ADAMTS-5	Mediated NO as inflammatory marker	[287]
Monoterpenes	α-pinene	UVA irradiation, mouse skin	**m′**	Skin photoaging	Improved oxidative damage and lipid peroxidation, inhibited iNOS, VEGF, TNF-α, IL-6, COX-2, NF-κB p65, Bax, Bcl-2, caspase-3, and caspase-9, suppressed MMP-2, -9, and -13 expressions	Inhibited iNOS as inflam-matory marker	[288]
Monoterpenes	Thymoquinone	Middle-aged rats	**n′**	Endothelial aging	Normalized expression of eNOS and Ca^2+^-activated K^+^ channels, angiotensin system and oxidative stress	Restored NO- and EDHF-mediated relaxation → ↓eNOS	[289,290]
Carotenoids	β-carotene	Ozone-induced aging in murine skin	**o′**	Skin photoaging	Downregulated the stimulation of MIP2, TNFα, iNOS, and HO-1	Downregulated iNOS as inflammatory marker	[291]
Carotenoids	Zeaxanthin	d-galactose-treated rats	**p′**	Aging-related cardiac dysfunctions	↓IL-6 and iNOS, ↑glucose transporter-4 and SOD, activated retinoid receptor-α in cardiac tissue	Downregulated iNOS as inflammatory marker	[292]
Carotenoids	Astaxanthin	LPS model of microglial cell stimulation	**q′**	Neuroinflammation during aging	Blocked COX-2 and iNOS	Inhibited iNOS as inflammatory marker, ↓NO production	[293]
Alkaloids	Higenamine	Human nucleus pulposus cells	**r′**	Aging-related intervertebral disc degeneration	Repressed NF-κB signaling pathway, ↓iNOS, NO, PGE2, COX-2, TNF-α, IL-6, MMP-3 and MMP-13, ADAMTS-4 and ADAMTS-5	Mediated NO and iNOS as inflammatory markers	[294]
Phenylpropanoid dibenzylbutyrolactone lignans (phyto-oestrogens)	Arctigenin	Human chondrocytes obtained from aged people with osteoarthritis	**s′**	Osteoarthritis	↓PGE2, COX-2, iNOS, NO, IL-6, and TNF-α, inhibited PI3K/Akt and NF-κB pathways	Mediated NO and iNOS as inflammatory markers	[295]
Organosulfur compounds	α-lipoic acid	Aged and high-fat diet-fed diabetic rats	**t′**	Endothelial aging	Restored endothelial function, ameliorated the oxidative damages, recoupled eNOS, ↑NO bioavailability	Recoupled eNOS, ↑NO bioavailability	[296]
Steroid saponins	Dioscin	H_2_O_2_-treated PC12 cells and D-galactose-induced aging rats	**u′**	Brain aging	Regulated MAPK and Nrf2/ARE pathways and adjusted content of oxidative damage and inflammatory markers, ameliorated memory and spatial learning, ↓levels of NOS in brain tissue	↓NOS attributed to reduction of oxidative stress	[297,298]
Sterols	Ergostatrien-3β-ol (EK100)	UVB-induced erythema, wrinkle creation, and epidermal thickness in the hairless mice skin	**v′**	Skin photoaging	Inhibited the expression of IL-6, MMP-1, iNOS, and NF-κB, ↓transepidermal water loss	Inhibited iNOS as inflam-matory marker	[299]
Organic isothiocyanates	Sulforaphane	d-galactose induced liver aging in rats	**w′**	Liver aging	Ameliorated liver biomarkers, ↓oxidative stress, ↓NO, protein carbonyl, TNF-α, and TGF-β, and prevented dysregulation of hepatic Nrf2/Keap1/HO-1	Mediated NO as an oxidative stress marker	[300]
Phenylethanosides	Forsythiaside	Brain homogenates of aged SAMP8 mice	**x′**	Memory deficits	↑Memory performance, ↓the levels of IL-1β, NO, MDA and NE levels, and ↑activities of T-SOD, GPx and GLU and ACh levels	Mediated NO as an oxidative stress marker	[301]
Indolamines	Melatonin	Aged rats	**y′**	Sepsis	↓Aging-promoted iNOS expression	Mediated iNOS as inflam-matory marker	[302,303]
PUFAs	Omega-3 (EPA:DHA 6:1)	Mesenteric artery	-	Endothelial aging	Restored endothelium-dependent NO-intervened relaxation, normalized angiotensin-converting enzyme (ACE)/angiotensin type 1 receptor (AT1R)/NADPH oxidase, ↓ROS production	Prevented upregulation of eNOS → restored endothelium-dependent NO-mediated relaxations	[304]
Probiotics	*Lactobacillus plantarum* LP-CQPC11	d-galactose induced aging in mice	-	Oxidation and aging	↑SOD, GSH-Px, and GSH, ↓NO and MDA in the serum, liver, and spleen, upregulated the expression of nNOS, eNOS, Mn-SOD, Cu/Zn-SOD, CAT, HO-1, y-glutamylcysteine synthetase, Nrf2, and NAD(P)H dehydrogenase [quinone] 1, upregulated SOD1, SOD2, CAT, GSH1, and GSH2 protein expression in mouse liver and spleen tissues	Restored the mRNA levels of nNOS, eNOS, and iNOS to normal → prevented oxidative stress	[305]
Amino-acids	l-arginine	HUVECs exposed to high levels of glucose	**z′**	Senescent changes	Suppressed the mitigation of eNOS and Akt activities	Akt–eNOS–NO pathway	[306]

## Data Availability

Not applicable.

## Abbreviations

Aβ	Amyloid-beta
BH_4_	(6r-)-tetrahydro-l-biopterin
cAMP	Cyclic adenosine monophosphate
cGMP	Cyclic guanosine monophosphate
CNS	Central Nervous System
COX	Cyclooxygenase
ecSOD	Extracellular superoxide dismutase
eNOS	Endothelial NOS
FAD	flavin adenine dinucleotide
GMP	guanosine monophosphate
GPx-1	guanosine monophosphate
iNOS	Inducible Nitric Oxide Synthase
MPF	M-phase-promoting factor
NADPH	nicotinamide-adenine-dinucleotide phosphate
NMDA	*N*-methyl-d-aspartate receptor
NO	Nitric Oxide
NOS	Nitric Oxide Synthase
PDE	phosphodiesterase
SOD	superoxide dismutase
TXA2	thromboxane A2
